# Dual-functional cationic hydrogel engineered for simultaneous prevention of postoperative tumor recurrence and wound infection

**DOI:** 10.1186/s12951-026-04490-3

**Published:** 2026-04-29

**Authors:** Yating Qin, Ke Yao, Yan Lin, Xinyue Li, Yifan Liu, Yilin Li, Yaping Li, Shuling Wang

**Affiliations:** 1 School of Pharmacy, Hangzhou Normal University, Hangzhou, 311121 China; 2 College of Chemical and Biological Engineering, Zhejiang University, Hangzhou, 311121 China

**Keywords:** Hydrogel, Tumor recurrence, Nanozyme, Elemene, Antibacterial

## Abstract

Postoperative recurrence and infection continue to pose major challenges after tumor resection. To address these, we developed a multifunctional nanocomposite hydrogel, designated as Gel@CAR-M@Mn@ELE. It is constructed by encapsulating CAR-M@Mn@ELE and immunomodulatory/antibacterial *Dendrobium officinale* polysaccharide (DOP) within a quaternary ammonium-modified gelatin methacryloyl hydrogel matrix. The CAR-M@Mn@ELE not only loads elemene (ELE) into porous Mn nanozymes (Mn NPs) but is also coated with EpCAM-targeting chimeric antigen receptor-engineered 293T cell membranes (CAR-M). Notably, through multiple modifications, the catalytic activity of Mn NPs has been preserved. In vitro studies demonstrated that CAR-M@Mn@ELE selectively targeted and killed 4T1 cancer cells (EpCAM^+^), while the hydrogel exhibited potent antibacterial activity against *Escherichia coli* (*E. coli*) and *Staphylococcus aureus* (*S. aureus*). In vivo evaluation using a post-tumor-resection mouse model confirmed that Gel@CAR-M@Mn@ELE significantly suppressed tumor recurrence and prolonged survival. The combination of bioactive components within the hydrogel modulated the immunosuppressive tumor microenvironment, thereby enhancing its immunotherapeutic efficacy. Additionally, in a bacterial wound infection model, the hydrogel effectively inhibited bacterial growth and accelerated wound healing. The Gel@CAR-M@Mn@ELE offers a novel and promising strategy, supported by experimental evidence, for concurrently addressing the critical post-surgical challenges of tumor recurrence and wound infection.

**Graphical Abstract:**

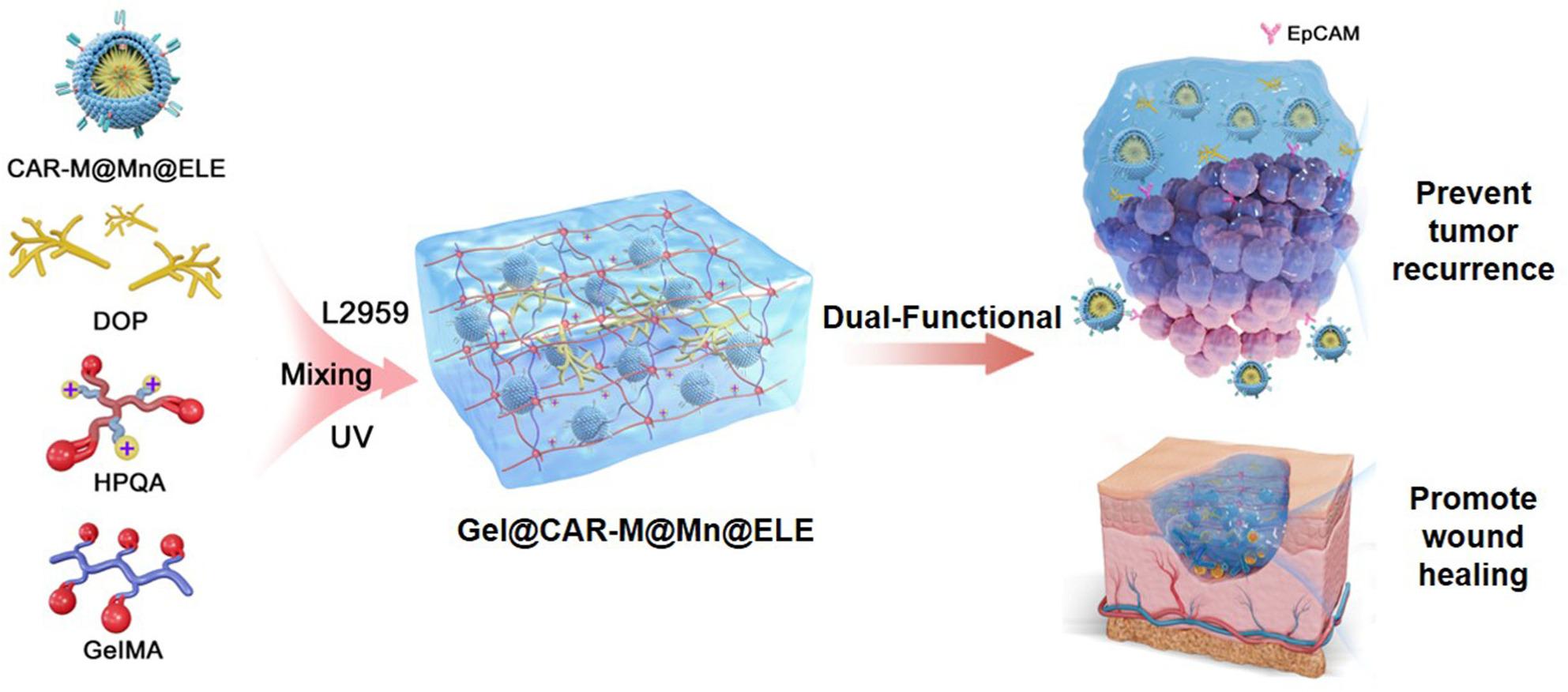

**Supplementary Information:**

The online version contains supplementary material available at 10.1186/s12951-026-04490-3.

## Introduction

Cancer continues to impose a substantial global health burden [1]. Surgery remains the main approach for treating solid tumors [2]. Although significant progress has been made in surgical techniques, there is often a presence of leftover tumor tissues and micrometastases at the resection margins, which notably elevates the chances of local recurrence and distant spread [3–5]. Additionally, postoperative wound infections represent a common clinical complication in oncological surgery, complicating patient recovery and potentially leading to systemic infections with life-threatening consequences [6, 7]. The pressing issues emphasized here underscore the immediate necessity for the creation of comprehensive treatment platforms capable of simultaneously tackling both the recurrence of tumors following surgery and bacterial infections.

Methacrylated gelatin (GelMA), a derivative of gelatin, has attracted considerable attention as a backbone material for in situ-forming hydrogels. GelMA preserves the low immunogenicity, bioadhesiveness, and biodegradability of gelatin, while its capacity to form stable, covalently crosslinked networks under ultraviolet light markedly enhances its mechanical robustness and thermal stability, making it an ideal matrix for postoperative applications [8–10]. Concurrently, cationic polymers, such as quaternary ammonium compounds (QACs) have been extensively explored for their potent and broad-spectrum antibacterial activity, which arises from their membrane-disrupting properties and low propensity to induce resistance [11–13]. By integrating QACs with GelMA hydrogels, a multifunctional platform can be constructed to synergistically tackle key postoperative challenges: localized drug delivery to suppress tumor recurrence, inherent antibacterial activity to prevent infections, and pro-healing properties to expedite wound repair. This integrated strategy leverages the complementary advantages of both materials, providing an effective postoperative delivery system for comprehensive post-surgical care.

Nanozymes, particularly manganese (Mn)-based ones, have garnered significant interest as alternatives to natural enzymes due to their cost-effectiveness, robust stability, and tunable catalytic activity [14, 15]. Their therapeutic potential arises from the ability to catalyze the generation of reactive oxygen species (ROS) from endogenous substrates, inducing oxidative stress and immunogenic cell death (ICD) in tumor cells. Furthermore, the concomitant release of Mn²⁺ serves as a potent immunostimulant by promoting dendritic cell (DC) maturation and macrophage M1 polarization, thereby orchestrating a robust antitumor immune response [16, 17]. However, the efficacy of such nanozymes is often compromised by non-specific distribution and insufficient tumor accumulation. To address this limitation, biomimetic coating with cell membranes has been explored to enhance the biocompatibility and targeting of nanomedicines. While a promising strategy, natural membranes often lack the precision required for specific tumor targeting. Inspired by the high specificity of chimeric antigen receptor (CAR) technology, we designed a novel nanoenzyme by coating CAR-modified cell membranes on the surface of Mn-based nanoenzymes. This CAR-modified membrane cloak endows the nanoenzyme with the ability to target tumors through membrane surface-specific antibodies [18–20]. Critically, we verified that this sophisticated bio-interface engineering preserves the intrinsic catalytic activity of the manganese nanozyme, ensuring that its ROS-generating and immunostimulatory functions remain intact for effective tumor eradication.

To synergize with and amplify this targeted catalytic immunotherapy, we incorporated elemene (ELE), a clinical anticancer agent derived from traditional Chinese medicine. ELE not only exerts direct chemotherapeutic effects but also complements the action of nanozymes by independently inducing ICD and repolarizing tumor-associated macrophages (TAMs) towards the M1 phenotype [21–27]. This strategic combination is expected to yield a powerful synergistic effect, enhancing tumor cell killing and immune activation. To further improve the local management of the microenvironment after surgery, we have selected *Dendrobium officinale* polysaccharides (DOP), a natural polysaccharide with immunomodulatory, anti-tumor and broad-spectrum antibacterial activities [28–30]. Studies have demonstrated that DOP exerts significant inhibitory effects against various common pathogenic bacteria, including *S. aureus* and *E. coli* [31]. Furthermore, due to the presence of hydroxyl groups in DOP, it is capable of forming hydrogen bonds with other polymers, which has facilitated its application in the preparation of bioactive hydrogels [32].

**Scheme 1:**
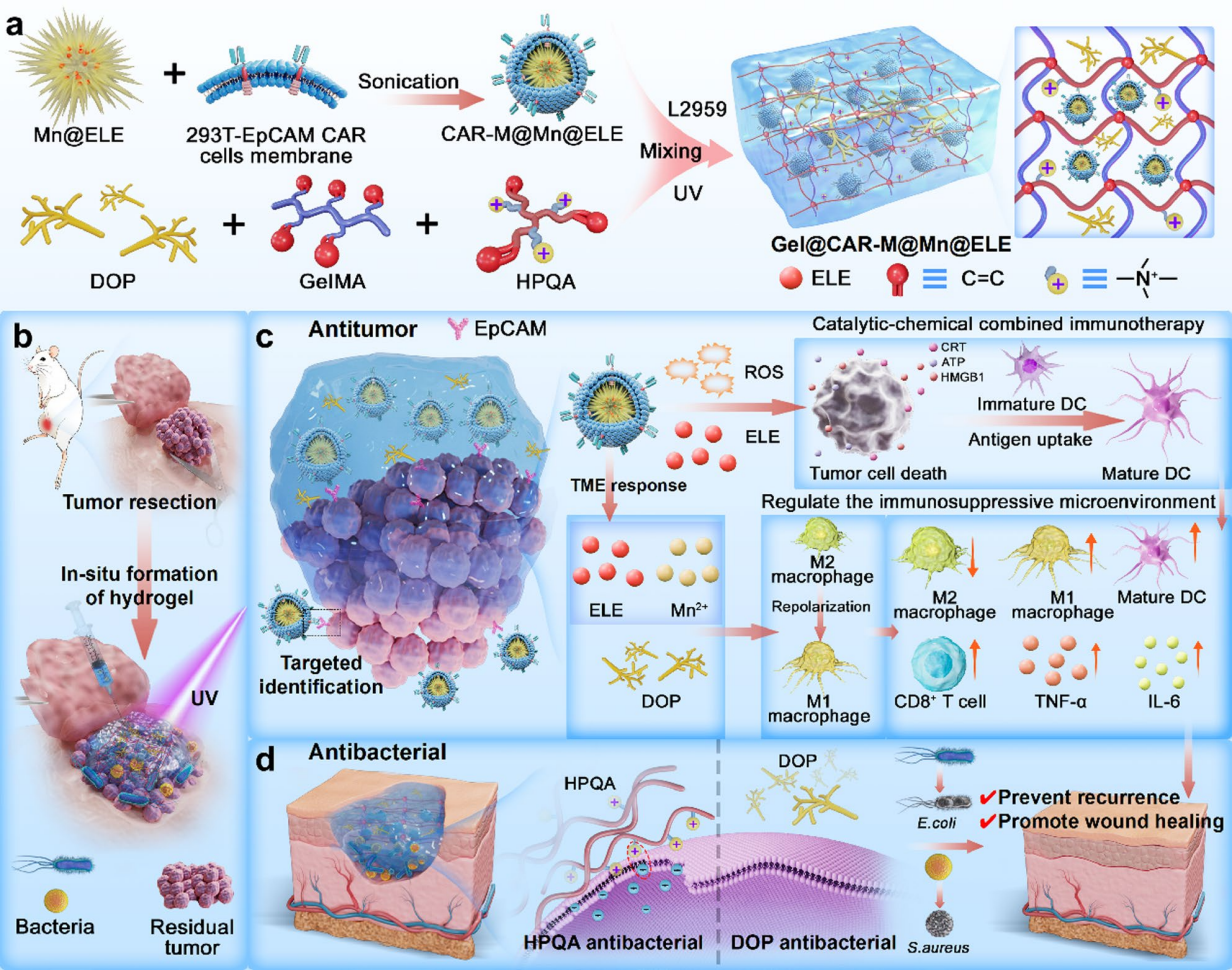
Schematic of Gel@CAR-M@Mn@ELE preparation and its dual function: preventing postoperative tumor recurrence and promoting antibacterial wound healing. (**a**) The preparation of CAR-M@Mn@ELE and Gel@CAR-M@Mn@ELE, emphasizing the composition of various functional components. (**b**) Schematic diagram of tumor resection and in situ gelation. (**c**) Antitumor via catalytic chemotherapy and TME modulation. (**d**) Infected wound healing enhanced by HPQA and DOP synergistic antibacterial action

In summary, we have rationally designed a multifunctional nanocomposite hydrogel (Gel@CAR-M@Mn@ELE) that integrates a tumor-targeting Mn nanozyme with the immunomodulatory agent ELE, the antibacterial polysaccharide DOP, and QACs into a unified platform for comprehensive post-surgical management. As illustrated in Scheme 1a, porous manganese-based nanozymes (Mn NPs) were first synthesized, and ELE was loaded into their pores via physical adsorption to form the Mn@ELE complex. This complex was subsequently cloaked with CAR-engineered cell membranes (CAR-M) to yield the actively targeted nanoplatform CAR-M@Mn@ELE, which specifically recognizes EpCAM antigens on tumor cells. These targeted nanoparticles were then uniformly embedded within a QACs-modified GelMA hydrogel matrix along with DOP. Importantly, the encapsulation process preserved the structural integrity and catalytic activity of the manganese nanozymes, enabling sustained ROS generation within the tumor microenvironment (TME). This hydrogel was formed in situ at the site of tumor resection after surgery (Scheme 1b). On one hand, CAR-M@Mn@ELE can specifically target EpCAM-positive (EpCAM⁺) 4T1 cancer cells and effectively suppress tumor recurrence. Meanwhile, the combination of bioactive components within the hydrogel modulates the immunosuppressive TME and enhances immunotherapy (Scheme 1c). On the other hand, QACs collaborate with DOP to produce a broad-spectrum antibacterial effect, efficiently inhibiting common pathogenic bacteria, preventing postoperative infections, and promoting wound healing (Scheme 1d). This multifunctional hydrogel integrates multiple active ingredients and collaboratively enhances the postoperative therapeutic effect on solid tumors, demonstrating significant potential for improving patient prognosis.

## Materials and methods

### Materials and reagents

Elemene was purchased from Dalian Jingang Pharmaceutical Co., Ltd. Fluorescein isothiocyanate isomer (FITC) was bought from Shanghai Yuanye Biotechnology Co., Ltd. 2-tert-butoxycarbonyl-2-methyl-3,4-dihydro-2 H-pyrrole-1-oxide (BMPO) was received from MedChemExpress. 2’,7’-dichlorodihydrofluorescein diacetate (DCFH-DA), DNA damage assay kit (γ-H2AX immunofluorescence) and live/dead bacterial staining kit (DMAO/PI) were purchased from Beyotime. MitoProbe JC-1 and 4% paraformaldehyde fix solution were purchased from Biosharp. All the fluorescently labeled antibodies were derived from Biolegend. D-Luciferin potassium salt and fluorescently labeled antibodies were bought from Shanghai Universal Biotech Co., Ltd. CCK-8, Elisa kits and other biological reagents were purchased from Beijing Solarbio Science & Technology Co., Ltd. All other chemical reagents were obtained from Energy Chemical.

### Preparation and characterization of CAR-M@Mn@ELE

Mn NPs were synthesized as reported [16]. Briefly, 100 mg KMnO₄ in 50 mL H₂O was stirred for 0.5 h. After adding 1 mL oleic acid, stirring continued for 5.5 h. The product was collected by centrifugation and washed with H₂O/ethanol. For ELE loading, 5 mg Mn NPs were dispersed in 5 mL ELE ethanol solution (0.3 mg/mL) and stirred for 12 h. After PBS washing, Mn@ELE was obtained. ELE loading capacity was determined by HPLC (Waters Alliance e2695, Waters, USA) [33]. Drug load (%) = (W_initial_ -W_remaining_) /W_Mn NPs_×100%, W_initial_ is the initial mass of ELE, W_remaining_ is the mass of ELE remaining in the supernatant, and W_Mn NPs_ is the mass of Mn NPs.

The 293T-EpCAM CAR-M was successfully prepared by lysing 293T-EpCAM CAR cells on ice using a mini handheld homogenizer [20]. The mixture of CAR-M and Mn@ELE was sonicated in an ice bath for 5 min using pulsed ultrasound in intervals of 5 s on and 5 s off [34, 35]. After centrifugation and PBS washing, the CAR-M@Mn@ELE was obtained. Its stability was assessed by measuring zeta potential in water and hydrodynamic size in RPMI-1640 + 10% FBS medium over time. Morphology was analyzed by SEM (Sigma 500, Zeiss, Germany) and TEM (JEM-F200, JEOL, Japan). Material characterization included: elemental composition via XPS (Thermo Kalpha, Thermo Fisher, America), surface area/pore size via BET (ASAP2460, Micromeritics, America), and optical properties via UV-vis (X-6, Metash, China). A Malvern Zetasizer (Zetasizer Lab, Malvern Panalytical, Britain) was used for size and zeta potential measurements.

###  In vitro TME-mimic elemene release test

The in vitro release of elemene was determined by dialysis. A TME-mimicking PBS solution (pH 6.5, 100 µM H₂O₂, 2 mM GSH, 0.5% SDS) [16] and a control PBS (pH 7.4, 0.5% SDS) were used as release media. The quantity of elemene released was measured using UV-vis.

### Detection of ROS types generated by CAR-M@Mn@ELE

ESR (EMXplus-6/1, Bruker, Germany) measurements used DMPO and BMPO to trap •OH and •O₂⁻, respectively. H₂O₂ (10 mM) was added to PBS containing CAR-M@Mn@ELE (10 µg/mL) at pH 6.5 or 7.4. A control contained H₂O₂ in PBS (pH 6.5) alone. The reaction systems were subsequently mixed with DMPO and BMPO for the specific detection of ^•^OH and ^•^O_2_^–^.

### Detection of the capability of GSH depletion

CAR-M@Mn@ELE (20 µg/mL) was mixed with GSH (1 mM) in PBS (pH 5.5) at 37 °C. At specified times, samples were taken, treated with DTNB (2 mM), and absorbance at 412 nm was measured by UV-vis.

### Cellular uptake

FITC-labeled Mn@ELE was prepared by stirring Mn@ELE with FITC at 4 °C for 12 h, followed by PBS washing. Coating with 293T-EpCAM CAR membrane produced FITC-labeled CAR-M@Mn@ELE. For cellular uptake, 4T1 (EpCAM⁺) cells were treated with either FITC-labeled Mn@ELE or CAR-M@Mn@ELE (25 µg/mL) for 1–2 h. MDA-MB-231 (EpCAM⁻) cells were also incubated with FITC-labeled CAR-M@Mn@ELE (25 µg/mL) for 1 h. Nuclei were stained with DAPI. Uptake was visualized by confocal laser scanning microscopy (CLSM, Waters Alliance e2695, Waters, America). The uptake of Mn@ELE and CAR-M@Mn@ELE by 4T1 cells after co-incubation for 2 h was further detected by flow cytometry (Beckman Coulter, CytoFLEX 5, America). Furthermore, to investigate the involvement of EpCAM in the internalization process, 4T1 cells were pre-incubated with an anti-EpCAM antibody to block surface EpCAM prior to co-cultivation with FITC-labeled CAR-M@Mn@ELE (25 µg/mL). After co-incubation for 1 h, the uptake was observed with CLSM.

### Cell viability assay

4T1 cells in 96-well plates were treated with different concentrations of Mn NPs, Mn@ELE, or CAR-M@Mn@ELE for 24 h. Cell viability was then assessed using CCK-8, and absorbance at 450 nm was measured with a microplate reader (Spark, Tecan, Switzerland).

### Detection of ROS and GSH levels in 4T1 cells

4T1 cells were treated with Mn@ELE or CAR-M@Mn@ELE (30 µg/mL) for 4 h to assess ROS or 24 h for GSH. Cells were then stained with DCFH-DA (10 µM, 30 min) for ROS or ThiolTracker Violet (10 µM, 1 h) for GSH, followed by DAPI nuclear staining. Intracellular ROS and GSH levels were visualized using CLSM. Meanwhile, the ROS level was further quantified by flow cytometry.

### Detection of mitochondrial integrity and DNA damage in 4T1 cells

4T1 cells in confocal dishes were treated with Mn@ELE or CAR-M@Mn@ELE (40 µg/mL). Mitochondrial membrane potential was evaluated using a JC-1 kit, and DNA damage was assessed via γ-H2AX immunofluorescence.

### Detection of ICD in 4T1 cells

ICD marker release (ATP, CRT, HMGB1) was assessed post-treatment. For ATP, 4T1 cells in 96-well plates were treated with Mn NPs, Mn@ELE, or CAR-M@Mn@ELE (40 µg/mL) for 24 h, and extracellular ATP was quantified. For CRT and HMGB1, treated cells in confocal dishes were fixed, permeabilized, blocked, and incubated overnight at 4 °C with respective primary antibodies. After applying fluorescent secondary antibodies and DAPI, levels were visualized by CLSM.

### Preparation and characterization of Gel@CAR-M@Mn@ELE

GelMA was synthesized following literature methods [9, 36]. Briefly, gelatin (1 g) was dissolved in water (10 mL) at 50 °C, followed by reaction with methacrylate anhydride (1.2 g) for 4 h. The product was purified by dialysis. The quaternary ammonium salt hyperbranched polymers (HPQA) was synthesized by copolymerizing polyethylene glycol diacrylate (PEGDA) and acryloyloxyethyltrimethyl ammonium chloride (DAC) in DMSO using tetraethyl thiuram disulfide (DS) and 2,2’-azobis(2-methylpropionitrile) (AIBN) as initiators under nitrogen. The reaction mixture was degassed, heated to 70 °C for 7 h, and dialyzed to obtain the purified polymer. The structures of GelMA and HPQA were confirmed by ¹H NMR (Avance-400, Bruker, America) and ATR-IR spectroscopy (IS50, Thermo Fisher Scientific, America).

Five hydrogels were prepared. G1 (Gel without DOP): GelMA (100 mg) and HPQA (100 mg) were dissolved in 1 mL water with 1% (w/v) photoinitiator 2959 at 40 °C, then UV-crosslinked (365 nm, 2 min). G2 (Gel): Prepared as G1 with added DOP (50 mg). G3 (Gel@CAR-M@Mn@ELE): GelMA (200 mg), HPQA (200 mg), and DOP (100 mg) were dissolved in 1 mL water with 1% photoinitiator 2959 at 40 °C, mixed with an equal volume of CAR-M@Mn@ELE solution (2 mg/mL), and UV-crosslinked. Gel@Mn@ELE (with Mn@ELE) and Gel@Mn (with Mn NPs) were synthesized similarly to G3, using the corresponding nanoparticles. Hydrogel structure was examined by SEM (Carl Zeiss, Germany), and rheological properties were characterized using a rheometer (HR20, TA Instruments, America).

### Evaluation of postoperative antitumor activity of Gel@CAR-M@Mn@ELE

A subcutaneous tumor model was established by injecting 4T1-Luc cells (6 × 10⁶) into BALB/c mouse hind legs. When tumors reached ~ 200 mm³, mice were randomized into five groups (*n* = 5) for surgical resection, simulating residual disease. Different hydrogel precursor solutions were injected into the cavity and cured in situ with UV light. Groups: (Ⅰ) no treatment as control, (Ⅱ) Gel (DOP: 5 mg), (Ⅲ) Gel@Mn (DOP: 5 mg; Mn NPs: 100 µg), (Ⅳ) Gel@Mn@ELE (DOP: 5 mg; Mn@ELE: 100 µg) and (Ⅴ) Gel@CAR-M@Mn@ELE (DOP: 5 mg; CAR-M@Mn@ELE: 100 µg). Tumor recurrence was monitored via in vivo imaging on days 0, 10, and 20, and body weights were recorded. When the recurrence volume of the tumor exceeds 2000 mm³ or the survival time exceeds 45 days, the mice will be euthanized. Tumor tissues were harvested for immune analysis. Using the previously described incomplete resection model (3 mice per group), single-cell suspensions were prepared from tumor tissues on day 9 via enzymatic digestion. Immune cell populations were analyzed by flow cytometry using fluorescent antibodies: CD8⁺ T cells (CD3⁺CD8⁺), mature DCs (CD11c⁺CD80⁺CD86⁺), and macrophages (M1: CD11b⁺F4/80⁺CD80⁺; M2: CD11b⁺F4/80⁺CD206⁺). Serum tumor necrosis factor-α (‌TNF-α) and interleukin-6 (IL-6) levels were measured by ELISA. Tumor proliferation was assessed by Ki67 immunohistochemistry. CD8⁺ T cell infiltration was compared between control and Gel@CAR-M@Mn@ELE groups via Immunohistochemical (IHC).

### In vivo antibacterial and wound repair of Gel@CAR-M@Mn@ELE

A *S. aureus* wound infection model was established in BALB/c mice by creating a back wound inoculated with 20 µL of bacteria (1 × 10⁸ CFU/mL). Mice were randomized into four groups: Control, Gel without DOP, Gel and Gel@CAR-M@Mn@ELE. All gels were formed in situ after being exposed to 365 nm UV light for 2 min. Wound areas were photographed and measured using ImageJ on days 0, 3, 7, 9, and 11. Healing rate was calculated as: Healing rate (%) = (A_0_-A_R_)/A_0_ × 100%, where A_0_ represents the area of the original wound on day 0 and A_R_ represents the area of the wound on day R. To evaluate the antibacterial efficacy, bacterial samples were collected from the wounds on days 3 and 5 by applying 50 µL of sterile saline onto the wound surface. Then, 5 µL of the bacterial suspension was inoculated onto LB agar plates and incubated at 37 °C for 20 h. Images of the colonies were captured, and the colonies were counted. The bactericidal rate was calculated using the following formula: Bactericidal rate (%) = (CFU of control group-CFU of experimental group)/CFU of control group×100%. On day 12, mice were euthanized and periwound tissues were collected for H&E and Masson staining.

### Cell lines and animals

The 293T-EpCAM CAR cell line were purchased from Vigen Biotechnology (Zhenjiang) Co., Ltd. The L929 and MDA-MB-231 cell lines were purchased from Guangzhou Yuanjun Biotechnology Co., Ltd. The 293T-EpCAM CAR and L929 cell lines cultured in DMEM medium containing 10% fetal bovine serum and 1% penicillin-streptomycin. The MDA-MB-231 cell line was cultured in a high-glucose DMEM medium containing 10% fetal bovine serum and penicillin streptomycin. The 4T1 and 4T1-luc cell lines were purchased from Shanghai Kanglang Biotechnology Co., Ltd., and cultured in 1640 medium containing 10% fetal bovine serum and 1% penicillin-streptomycin. BALB/c mice aged 6 to 8 weeks were purchased from the Animal Center of Hangzhou Normal University.

### Statistical method

All data were presented as mean ± standard deviation (SD). Unless otherwise specified more than three times, the experiments were conducted three times independently. The average fluorescence intensity was quantified using ImageJ software. Statistical analysis was performed using GraphPad Prism 9.5 software. Survival curves were compared using the log-rank test; other data were analyzed with one-way or two-way ANOVA. Statistical significance was indicated by an asterisk, where *P < 0.05; **P < 0.01; ***P < 0.001; ****P < 0.0001.

## Result and discussion

### Preparation and characterization of CAR-M@Mn@ELE

**Fig. 1:**
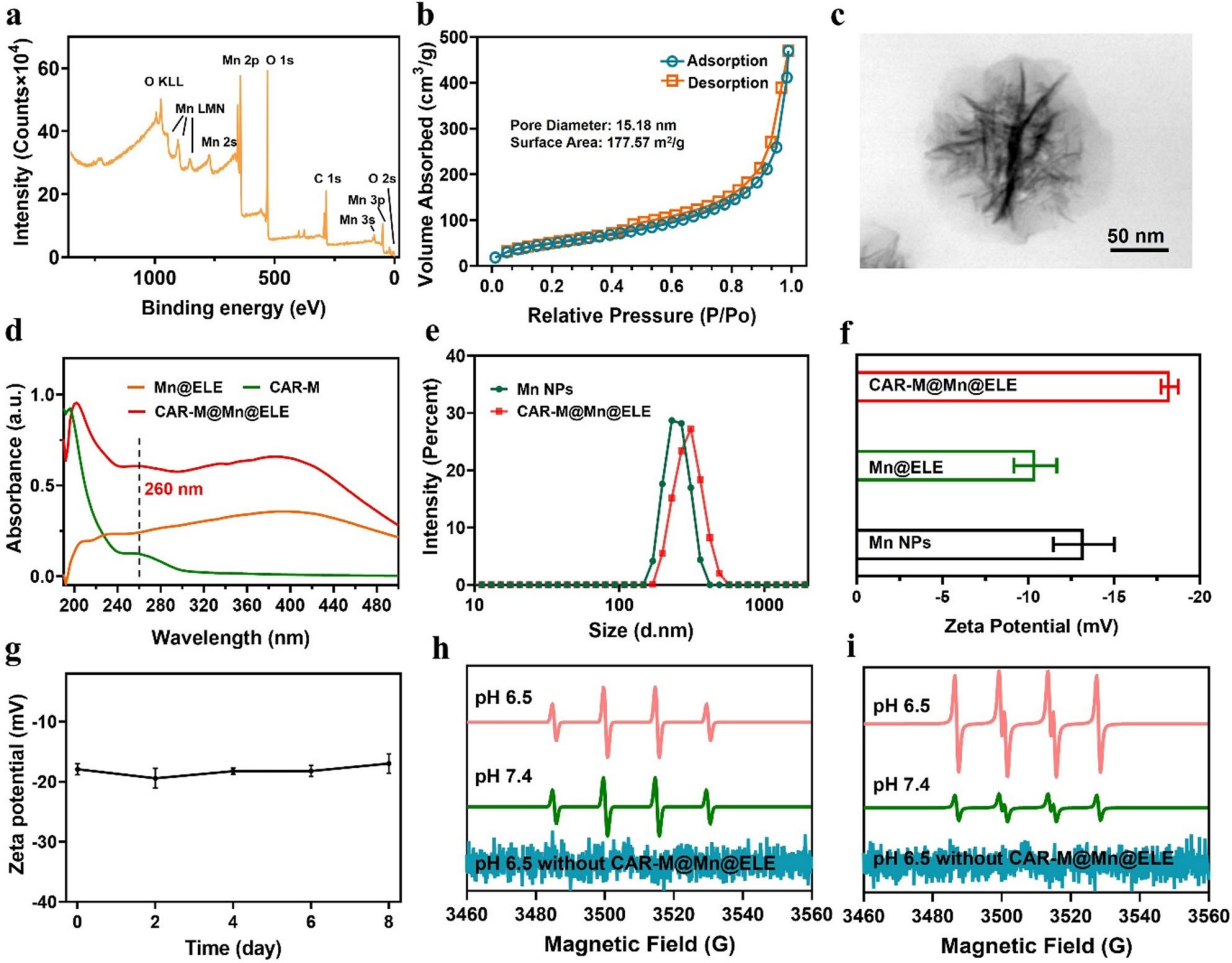
Characterizations of Mn NPs, Mn@ELE and CAR-M@Mn@ELE. (**a**) Full-range survey XPS spectrum for Mn NPs. (**b**) N_2_ adsorption-desorption isotherms and pore size distribution of Mn NPs. (**c**) Representative TEM image of CAR-M@Mn@ELE. (**d**) UV-vis spectra of the Mn@ELE, CAR-M and CAR-M@Mn@ELE. (**e**) The hydrodynamic size and (**f**) The zeta potential of Mn NPs, Mn@ELE and CAR-M@Mn@ELE (Concentration: 0.125 mg/ml, Solvent: Deionized water). (**g**) The zeta potential of CAR-M@Mn@ELE (Concentration: 0.125 mg/ml, Solvent: Deionized water) for different durations. ESR spectra of •OH (**h**) and •O^2−^ (**i**) under different conditions

Mn NPs exhibiting enzyme-like catalytic activity were synthesized. The SEM analysis demonstrated that the Mn NPs possess a porous, flower-like spherical structure with an average particle size of 124.9 ± 3.25 nm (Figure S1). XPS analysis confirmed the presence of Mn and O elements in the Mn NPs (Fig. 1a), revealing mixed valence states (Mn⁴⁺, Mn³⁺, and Mn²⁺) through characteristic peaks (Figure S2a). The lower valences (Mn³⁺ and Mn²⁺) confer peroxidase (POD) and oxidase (OXD)-like activities, while Mn⁴⁺ contributes to glutathione (GSH) depletion, enabling multi-enzyme functionality. Figure S2b presents the XPS spectrum characterizing the chemical state of oxygen in the Mn NPs. N₂ adsorption-desorption analysis indicated a high surface area (177.57 m²/g) and pore size (15.18 nm), which is suitable for the loading of ELE (Fig. 1b). Successful loading of ELE to form Mn@ELE was confirmed by UV-vis (Figure S3), yielding a loading capacity of 21.46 ± 0.95% (wt/wt) by HPLC. For tumor targeting, Mn@ELE was coated with EpCAM-targeting CAR-M, forming CAR-M@Mn@ELE. TEM revealed a core-shell flower-like structure (140 ± 4.62 nm, Fig. 1c), and elemental mapping (N distribution) confirmed the presence of CAR-M coating (Figure S4). Coating was further confirmed by UV-vis (Fig. 1d) and increased size/negative zeta potential (Fig. 1e, f and S5). CAR-M@Mn@ELE maintained stability in size and zeta potential (Fig. 1g and S6). Under TME-mimicking conditions (PBS, pH 6.5, 100 µM H_2_O_2_, 2 mM GSH, 0.5% SDS), CAR-M@Mn@ELE exhibited a significantly enhanced ELE release (54.26 ± 1.67%) versus control (pH 7.4, 0.5% SDS), thereby confirming TME-responsive drug release (Figure S7). Its mixed valences conferred catalase (CAT), peroxidase (POD) and oxidase (OXD)-like activities (Figure S8-10) and depleting GSH via Mn⁴⁺ redox (Figure S11). ESR analysis further elucidated the pH dependence of these activities (Fig. 1h and i). Specifically, the •OH signal (POD-like activity) remained largely unchanged from pH 7.4 to 6.5. In contrast, the •O₂⁻ signal (OXD-like activity) was markedly intensified at pH 6.5, confirming its pronounced pH sensitivity. In summary, the above assay results have demonstrated the successful synthesis of the CAR-M@Mn@ELE, which integrates synergistic therapeutic capabilities including targeted drug delivery, enzyme catalysis, and GSH depletion. This multifunctional platform presents a promising strategy for the advancement of postoperative combination therapies aimed at preventing tumor recurrence.

### Preparation and characterization of Gel@CAR-M@Mn@ELE

**Fig. 2:**
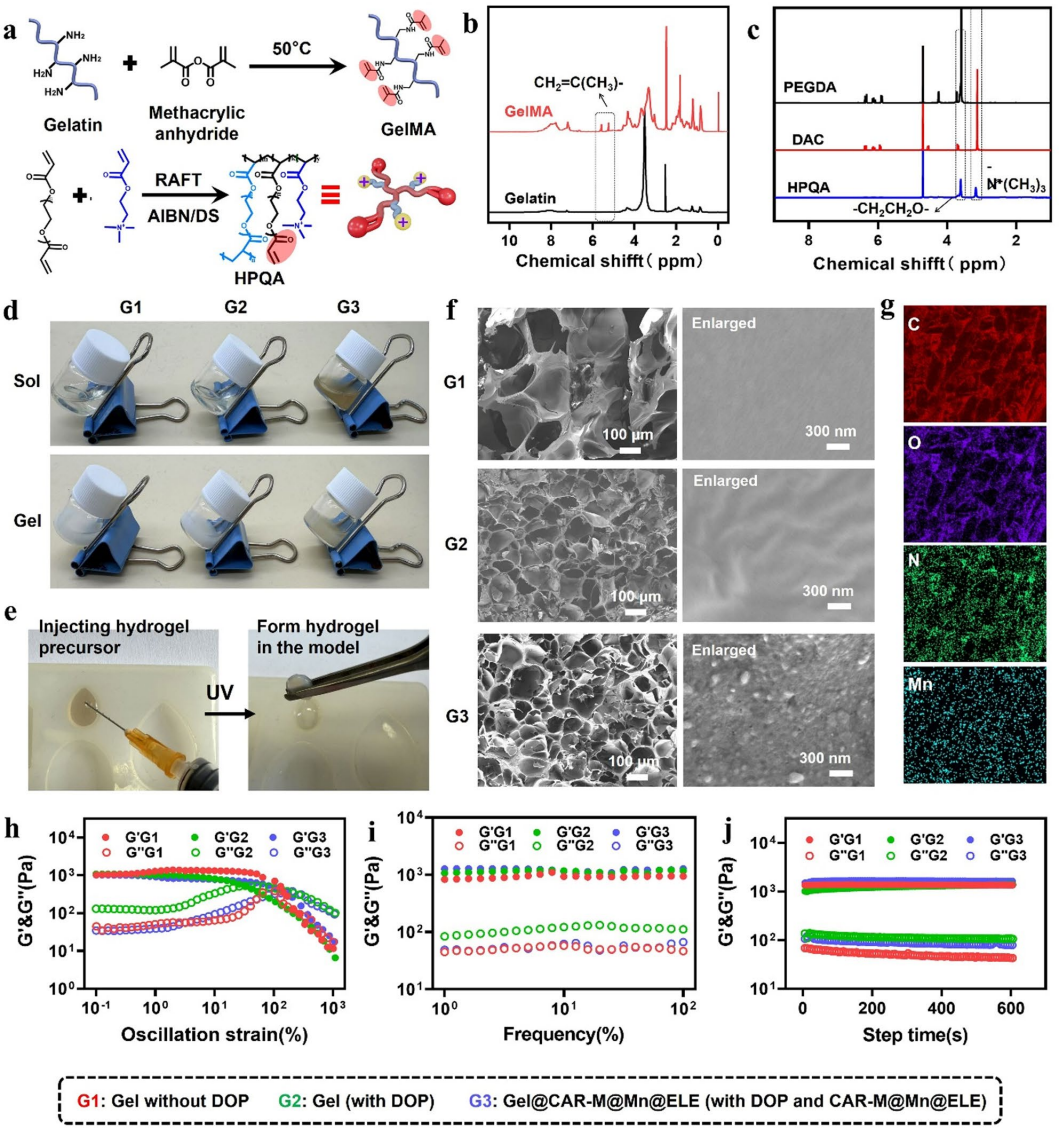
Characterization of Gel@CAR-M@Mn@ELE. (**a**) A schematic diagram illustrating the synthesis of GelMA and HPQA. (**b**) ^1^H-NMR profiles of GelMA and Gelatin. (**c**) ^1^H-NMR profiles of PEGDA, DAC, and HPQA. (**d**) Photographs of the sol and gel states for G1, G2 and G3. (**e**) A process diagram depicting the formation of a solid gel under ultraviolet light induction after injecting the hydrogel precursor solution of G3 into the mold. (**f**) SEM images of G1, G2 and G3. (**g**) Element distribution of G3. Strain scanning (**h**), dynamic frequency scanning (**i**) and time scanning (**j**) result diagram of the G1, G2 and G3. (G1: Gel without DOP; G2: Gel with DOP; G3: Gel@CAR-M@Mn@ELE with DOP and CAR-M@Mn@ELE)

The construction of Gel@CAR-M@Mn@ELE began with synthesizing GelMA and HPQA. GelMA was prepared by reacting gelatin with MA (Fig. 2a). The successful preparation of GelMA was confirmed by ^1^H NMR spectroscopy (Fig. 2b), where new peaks at δ = 5.2 and 5.5 ppm, corresponding to the characteristic vinyl protons (CH_2_=C(CH_3_)–) of the methacryloyl group, appeared in the GelMA spectrum compared to that of gelatin. This observation confirms the successful grafting of methacryloyl groups onto the gelatin molecule. HPQA was synthesized through the reaction of PEGDA and DAC. In the ^1^H NMR spectrum of HPQA (Fig. 2c), the characteristic peak at δ = 3.1 ppm is attributed to the methyl protons of the quaternary ammonium group (–N^+^(CH_3_)_3_). The ATR-IR spectrum (Figure S12) displayed characteristic absorptions at 1473 cm^− 1^, which are attributed to the C–N stretching vibration of the quaternary ammonium group (–NR_4_^+^), and at 2872 cm^− 1^, corresponding to the C–H stretching vibration of methylene groups (–CH_2_–) within the PEG segment. Collectively, these spectroscopic results confirm the successful synthesis of HPQA. Three hydrogels were synthesized: G1 (Gel without DOP, GelMA/HPQA), G2 (Gel, GelMA/HPQA with DOP), and G3 (Gel@CAR-M@Mn@ELE, GelMA/HPQA with DOP and CAR-M@Mn@ELE). All underwent UV crosslinking, exhibiting sol-gel transition (Fig. 2d) and injectable precursor gelation (Fig. 2e). This injectable characteristic, combined with photoinitiated crosslinking, facilitates in situ hydrogel formation on wound surfaces following tumor resection, underscoring its clinical applicability. The lyophilized hydrogels of G1, G2, and G3 exhibited a porous structure as evidenced by SEM results, which is advantageous for drug delivery (Fig. 2f). Low-magnification images revealed three-dimensional network structures in all hydrogels, with G2 and G3 exhibiting denser networks compared to G1. This structural compaction can be attributed to the incorporation of DOP, which enhances crosslinking density through interchain hydrogen bonding. Higher-magnification SEM of G3 revealed rougher pore walls and uniformly dispersed particulate features when compared to G1 and G2, indicating successful embedding of CAR-M@Mn@Mn@ELE nanoparticles. Elemental mapping (Fig. 2g) confirmed the distribution of Mn throughout the G3 matrix, thereby further verifying the incorporation of nanoparticles. Gel strength measurements (Figure S13) yielded values of 983.15 Pa (G1), 1088.50 Pa (G2), and 1216.09 Pa (G3), demonstrating mechanical compatibility with wound tissue. Strain amplitude sweeps (Fig. 2h) revealed solid-to-liquid transitions upon exceeding the G’/G” crossover point. G3 exhibited the highest critical strain (170%), indicating superior structural stability. Frequency and time sweeps (Fig. 2i and j) confirmed elastic dominance; G2 exhibited higher G”, suggesting enhanced viscous dissipation from polysaccharide-DOP interaction. Furthermore, the release of CAR-M@Mn@ELE from G3 was demonstrated in an in vitro simulation of the postoperative microenvironment (Figure S14). In summary, the developed hydrogel integrates uniform CAR-M@Mn@ELE distribution, catalytic activity, suitable strength, and stable rheology, supporting its potential for postoperative tumor therapy.

###  In vitro antitumor efficacy of CAR-M@Mn@ELE

**Fig. 3:**
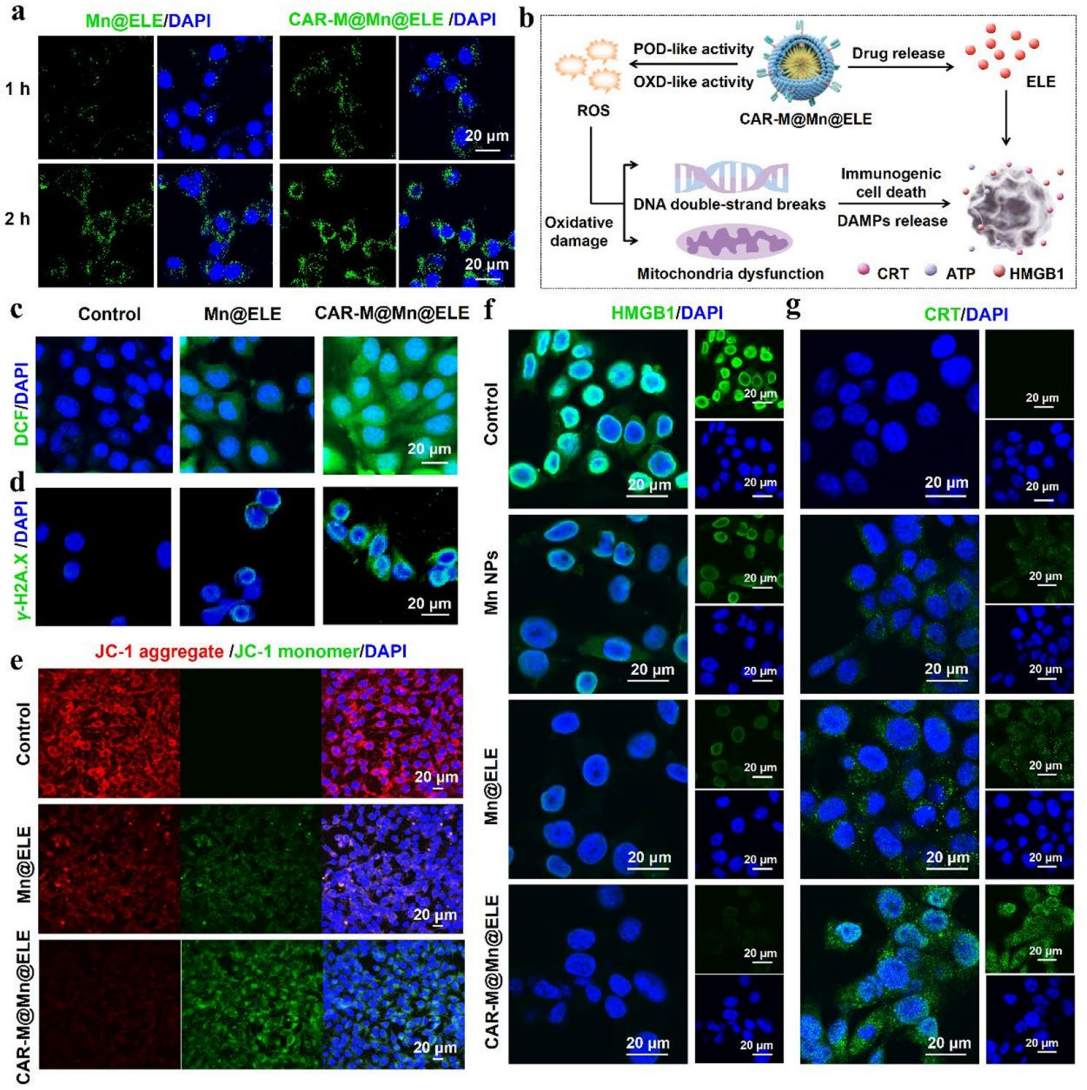
Intracellular behaviors of CAR-M@Mn@ELE. (**a**) CLSM images of 4T1 cells at various time points following co-incubation with Mn@ELE and CAR-M@Mn@ELE, respectively. (**b**) Schematic representation of ICD induced by CAR-M@Mn@ELE. Analysis of total ROS production (**c**) and γ-H2A.X expression levels (**d**) in 4T1 cells after co-incubation with Mn@ELE and CAR-M@Mn@ELE. (**e**) Assessment of mitochondrial membrane potential (ΔΨm) alterations in 4T1 cells following treatment with Mn@ELE and CAR-M@Mn@ELE, respectively. Expression levels of HMGB1 (**f**) and CRT (**g**) in 4T1 cells after co-incubation with Mn NPs, Mn@ELE, and CAR-M@Mn@ELE, respectively. Scale bar: 20 μm

The tumor-targeting efficacy of CAR-M@Mn@ELE was evaluated in EpCAM⁺ 4T1 cells. CLSM showed time-dependent cellular uptake, with significantly higher fluorescence intensity for FITC-labeled CAR-M@Mn@ELE than for FITC-labeled Mn@ELE at all time points, confirming CAR-M mediated targeting (Fig. 3a and S15). Flow cytometry analysis yielded consistent results (Figure S16). The significantly reduced uptake in both EpCAM^−^ MDA-MB-231 cells (Figure S17a) and EpCAM-blocked 4T1 cells (Figure S17b) further confirmed that the tumor-targeting capability of CAR-M@Mn@ELE nanoparticles was specifically driven by the EpCAM antigen. Furthermore, the targeting property of the CAR-M@Mn@ELE released from Gel@CAR-M@Mn@ELE has also been demonstrated (Figure S18). CCK-8 assays revealed concentration-dependent cytotoxicity for all nanoparticles (Figure S19). Mn@ELE was more cytotoxic than Mn NPs due to ELE synergy, and CAR-M@Mn@ELE showed the strongest effect, attributable to improved targeted uptake. CAR-M@Mn@ELE exhibited multi-enzyme catalytic activity that generated ROS and synergized with ELE to induce ICD in tumor cells (Fig. 3b). Intracellular ROS levels, detected by DCFH-DA, were significantly higher in both Mn@ELE and CAR-M@Mn@ELE groups, with the latter showing stronger fluorescence due to enhanced targeted uptake (Fig. 3c, S20 and S21). Furthermore, ThiolTracker Violet staining revealed that CAR-M@Mn@ELE depleted intracellular GSH more effectively than Mn@ELE, confirming its potent regulation of the tumor redox microenvironment (Figure S22). Elevated intracellular ROS levels can damage both DNA and mitochondria, leading to apoptosis. Immunofluorescence staining of γ-H2A.X (a DNA damage marker) revealed that CAR-M@Mn@ELE significantly increased DNA damage in 4T1 cells compared to both the control and Mn@ELE groups (Fig. 3d and S23a). Mitochondrial membrane potential (ΔΨm) was assessed using JC-1, which shifts from red (high ΔΨm) to green fluorescence (depolarized). CAR-M@Mn@ELE treatment sharply decreased red and increased green fluorescence versus other groups, indicating severe mitochondrial depolarization (Fig. 3e and S23b). These results confirm that CAR-M@Mn@ELE induces apoptosis via ROS-mediated DNA and mitochondrial damage. CAR-M@Mn@ELE induces immunogenic cell death (ICD) via damage-associated molecular patterns (DAMPs), including ATP secretion, CRT exposure, and HMGB1 translocation. It increased extracellular ATP by 6.26-fold (control: 1-fold), exceeding Mn NPs (2.92-fold) and Mn@ELE (3.60-fold) (Figure S24). Immunofluorescence showed progressive reduction of nuclear HMGB1, most pronounced with CAR-M@Mn@ELE (Fig. 3f and S25a), and maximal CRT surface exposure (Fig. 3g and S25b), attributable to ELE synergy and targeted delivery. In summary, CAR-M@Mn@ELE achieves precise EpCAM targeting, potent tumor killing, and effective ICD induction, highlighting its promise for postoperative therapy.

### Evaluation of postoperative antitumor activity of Gel@CAR-M@Mn@ELE

**Fig. 4:**
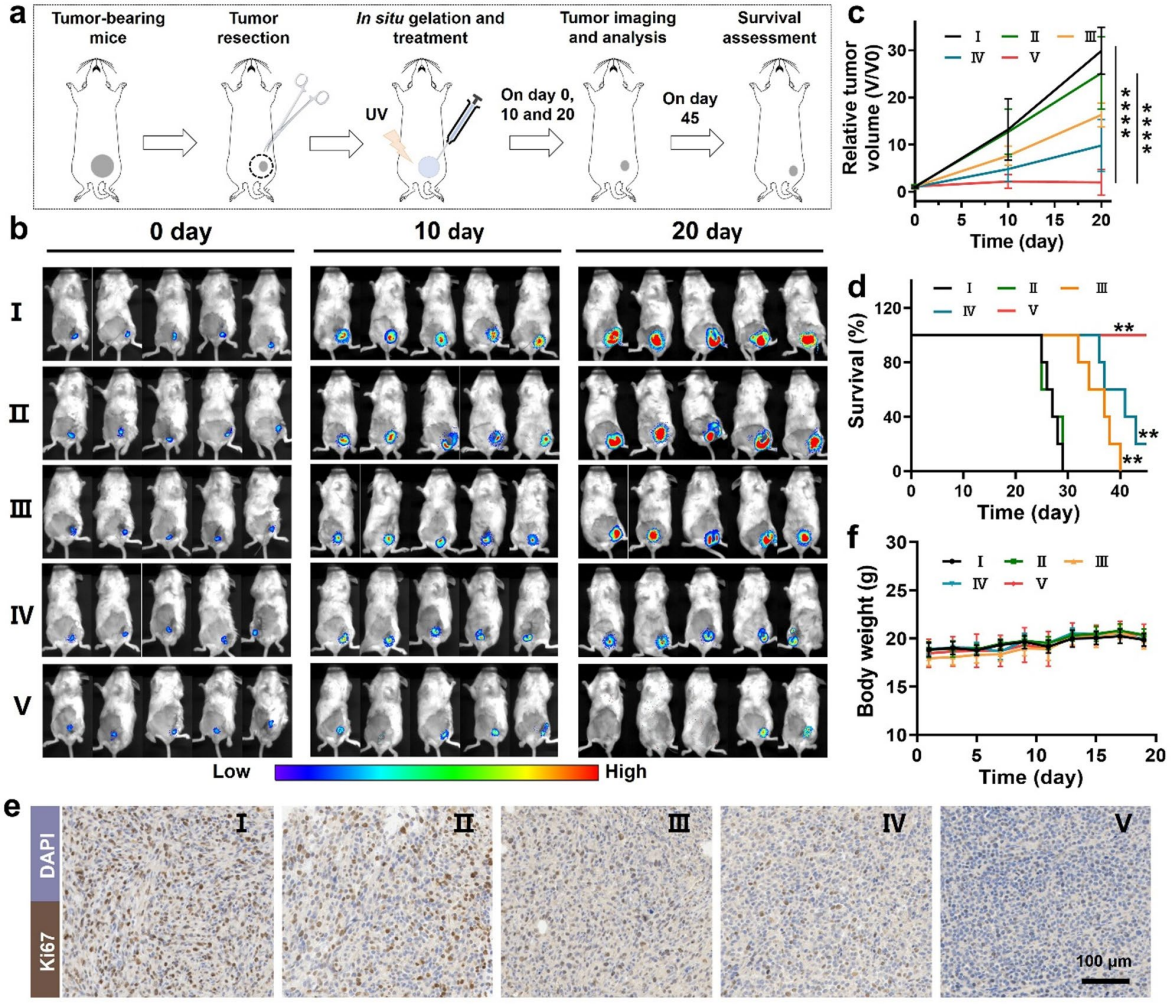
Efficacy of hydrogels in inhibiting tumor recurrence in vivo. (**a**) Construction of the 4T1-Luc tumor postoperative resection model and the schematic diagram of the in vivo treatment protocol. In vivo bioluminescence imaging of 4T1-Luc tumors (**b**) and corresponding tumor relative volume changes (**c**) across different treatment groups of mice following tumor surgery. (**d**) Survival curves among various treatment groups of mice post-tumor surgery. (**e**) Representative Ki67 immunohistochemistry staining of tumor tissues on day 9 after different treatment regimens. (**f**) Changes in body weight of mice across different treatment groups following tumor surgery. Ⅰ(Control), Ⅱ(Gel), Ⅲ(Gel@Mn), Ⅳ(Gel@Mn@ELE), Ⅴ(Gel@CAR-M@Mn@ELE). Scale bar: 100 μm. The data represent the mean ± SD. *n* = 5. **P < 0.01 and ****P < 0.0001

To assess hydrogel efficacy in preventing postoperative tumor recurrence, an incomplete resection model was established in mice using subcutaneous 4T1-Luc cells. Tumor progression was monitored via in vivo bioluminescence imaging (Fig. 4a). After 20 days, tumor volume in the Gel@CAR-M@Mn@ELE group increased only approximately 1.8-fold from post-resection, compared to approximately 14.8-fold (Gel@Mn), 9.8-fold (Gel@Mn@ELE), 29.7-fold (Control), and 22.2-fold (Gel alone) (Fig. 4b and c). The weak antitumor effect observed in the pure gel group may be attributed to the immunomodulatory properties of the DOP component present in the gel formulation [28–30]. Survival analysis showed that all mice in the Gel@CAR-M@Mn@ELE group survived to day 45, whereas other groups exhibited shorter survival (Fig. 4d). Ki67 staining on day 9 further confirmed the strongest therapeutic effect with Gel@CAR-M@Mn@ELE (Fig. 4e). The enhanced efficacy is attributed to the synergy between Mn NPs and ELE, combined with CAR-M targeting and sustained local release. Stable body weights indicated good biosafety (Fig. 4f).

**Fig. 5:**
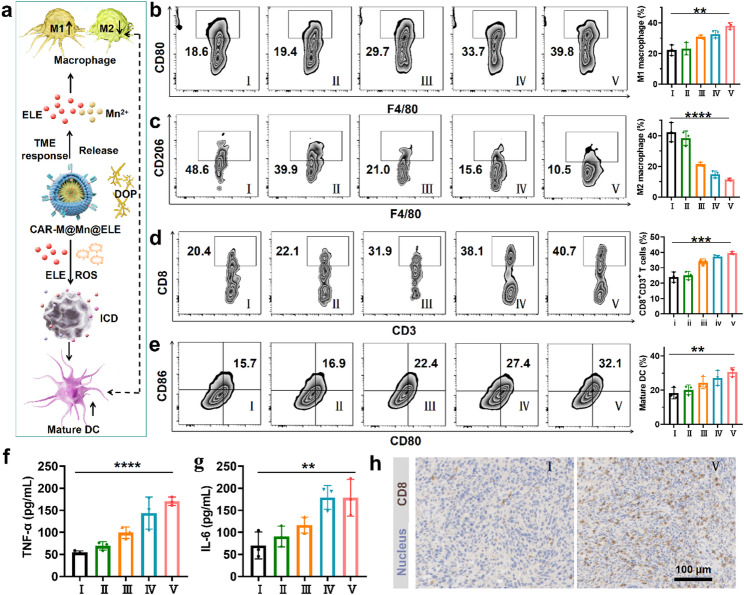
Antitumor immune response. (**a**) Schematic of antitumor immune response of CAR-M@Mn@ELE. Flow cytometry analysis of M1 (**b**) and M2 (**c**) macrophages, CD3^+^CD8^+^ T cells (**d**) and DC cells (**e**) in tumor tissues on day 9. ELISA analysis of TNF-α (**f**) and IL-6 (**g**) in serum from mice on day 9. (**h**) IHC analysis of CD3^+^CD8^+^ T cells infiltration in tumor sections collected on day 9. Ⅰ(Control), Ⅱ(Gel), Ⅲ(Gel@Mn), Ⅳ(Gel@Mn@ELE), Ⅴ(Gel@CAR-M@Mn@ELE). Scale bar: 100 μm. The data represent the mean ± SD, *n* = 3. **P < 0.01, ***P < 0.001 and ****P < 0.0001

In vitro experiments demonstrate that CAR-M@Mn@ELE enhances ICD. Furthermore, studies have indicated that Mn²⁺, ELE, and DOP individually can promote M2-to-M1 macrophage repolarization, while both Mn²⁺ and DOP can also directly induce the maturation of DC [16, 26–30]. Gel@CAR-M@Mn@ELE enhances antitumor immunity by remodeling the tumor immune microenvironment (Fig. 5a). On day 9 post-treatment, the M1/M2 macrophage ratio increased significantly in all nanoparticle-containing groups, most prominently with Gel@CAR-M@Mn@ELE (Fig. 5b and c). Similarly, proportions of cytotoxic T lymphocytes (CTLs, CD3⁺CD8⁺) and mature DCs were highest in the Gel@CAR-M@Mn@ELE group (Fig. 5d and e). This group also showed the greatest elevation in serum TNF-α and IL-6 levels (Fig. 5f and g) and increased CD8⁺ T cell infiltration (Fig. 5h). The superior efficacy of Gel@CAR-M@Mn@ELE stems from the synergy of Mn NPs and ELE, combined with CAR-M-mediated targeting and localized drug retention. The biosafety of Gel@CAR-M@Mn@ELE was further evaluated. The extracting solution showed no significant cytotoxicity to L929 cells after 24 h (Figure S26). The hemolysis rate of the extraction solution was determined to be 1.29%, indicating that the material exhibits non-hemolytic properties. In mice, 11 days post-implantation, no notable abnormalities were detected in key blood parameters (Figure S27) or major organ histology (Figure S28). Furthermore, Mn-based nanozymes are regarded as biodegradable and can be effectively eliminated from the body. These results confirm the good biocompatibility of Gel@CAR-M@Mn@ELE.

### Gel@CAR-M@Mn@ELE antibacterial and wound repair of evaluation

**Fig. 6:**
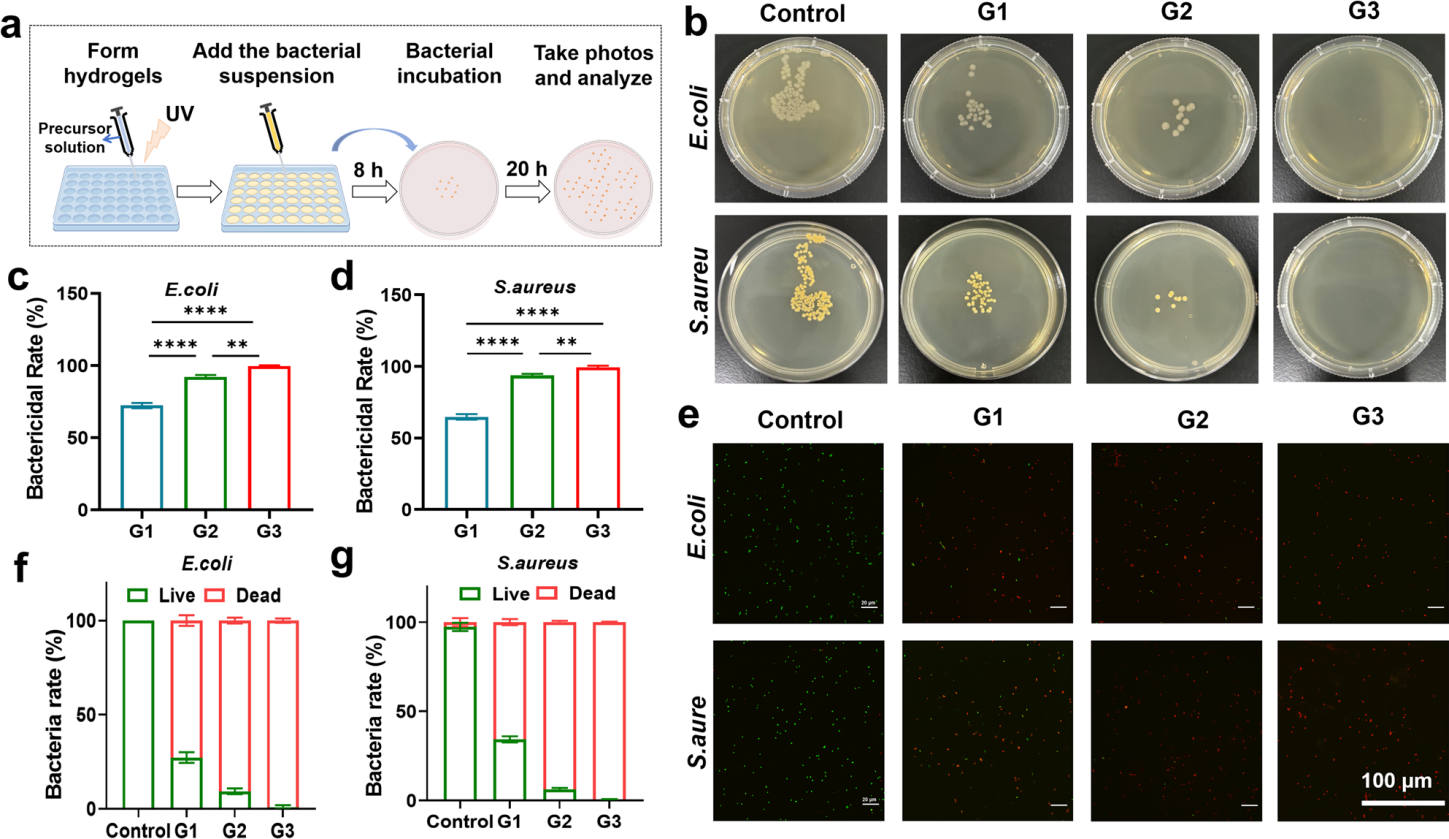
Antibacterial effects of hydrogels in vitro. (**a**) Schematic diagram of the antibacterial experiment of hydrogel. (**b**) Representative photographs of *S. aureus* and *E. coli* colonies after different treatments. Bactericidal rates of *S. aureus* (**c**) and *E. coli* (**d**) colonies after different treatments. (**e**) Representative DMAO/PI staining images of *S. aureus* and *E. coli* colonies after different treatments. The statistics of the live/dead of *S. aureus* (**f**) and *E. coli* (**g**) colonies after different treatments. Control: Normal saline; G1: Gel without DOP; G2: Gel with DOP; G3: Gel@CAR-M@Mn@ELE with DOP and CAR-M@Mn@ELE; Scale bar: 100 μm. The data represent the mean ± SD, n=3. **P < 0.01 and ****P<0.0001

The antibacterial efficacy of hydrogels G1, G2, and G3 was assessed against *E. coli* and *S. aureus* (Fig. 6a). Colony counting revealed significant reductions, with G1 achieving sterilization rates of 72.27 ± 1.8% (*E. coli*) and 64.68 ± 2.0% (*S. aureus*), attributed to HPQA (Fig. 6b-d). Both G2 and G3 exceeded 90% sterilization, demonstrating enhanced efficacy from incorporated DOP. DMAO/PI staining and quantitative fluorescence confirmed these results (Fig. 6e-g), showing over 90% bacterial mortality for G2 and G3. The enhanced red fluorescence is attributed to the influx of PI, which indicates compromised bacterial cell membrane integrity and increased membrane permeability. SEM imaging (Figure S29) further validated severe membrane damage and structural collapse in bacteria treated with G2 and G3, highlighting the synergistic antibacterial action of quaternary ammonium ions and DOP.

**Fig. 7:**
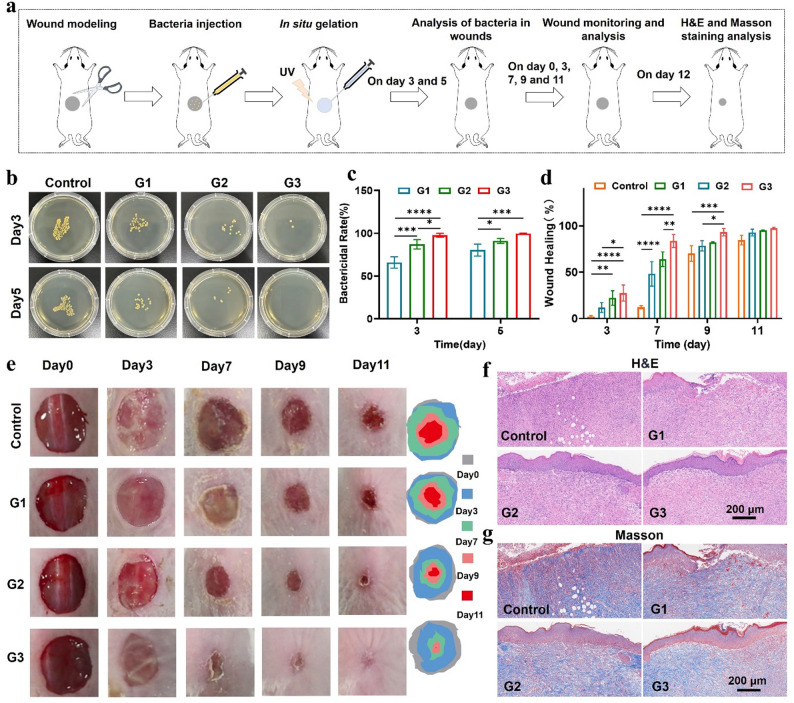
In vivo therapeutic effect of hydrogels for infected wound healing. (**a**) Schematic illustration of *S. aureus*-infected wound healing and analysis. The *S. aureus* colony plots (**b**) and the bactericidal rate of *S. aureus* (**c**) in each group of wounds at 3 and 5 days after treating the infectious wounds of mice with different hydrogels. The wound healing rate (**d**), representative photographs and traces of wounds (**e**) following treatment with various hydrogels at 0, 3, 7, 9, and 11 days post-treatment. H&E (**f**) and Masson (**g**) stainings of the wound healing regions of each group on day 12. Scale bar: 200 μm. The data represent the mean ± SD, *n* = 3. *P < 0.05, **P < 0.01, ***P < 0.001 and ****P < 0.0001

A murine model of *S. aureus*-infected skin wounds was utilized to assess the antibacterial efficacy of various hydrogels groups on infectious wounds and their therapeutic effects on promoting wound healing (Fig. 7a). Bacterial counts from wounds on days 3 and 5 post-treatment showed that all hydrogels (G1, G2, G3) significantly reduced colonies versus control, with G2 and G3 achieving > 90% reduction by day 5 (Fig. 7b and c). It can be seen that the hydrogel effectively controlled the bacterial infection at the wound site. Furthermore, the wound healing was accelerated in all treatment groups. G3 showed the highest healing rate (84.44 ± 3.1% by day 7, 95.81 ± 2.1% by day 9), with near-complete closure by day 11 (Fig. 7d and e). As is well known, once the infection is resolved, the wound will transition from the initial inflammatory/anti-infection stage to the tissue remodeling stage. On the 12th day after treatment, histological analysis (Fig. 7f, g and S30) revealed that the density of inflammatory cells in the treatment group decreased, the degree of re-epithelialization increased, and the deposition of collagen also risen. This indicates that the tissue was effectively repaired after the infection was controlled. This tissue regeneration effect is achieved by utilizing the initially necessary bactericidal and pro-inflammatory to control the infection. Our future research will focus on elucidating the mechanism of transition from the anti-infection stage to the regeneration stage to comprehensively understand and optimize the wound healing process.

## Conclusion

In summary, we developed a dual-functional cationic hydrogel, Gel@CAR-M@Mn@ELE, that effectively inhibits postoperative tumor recurrence and facilitates the healing of infected wounds. Its key advantages are as follows: (1) synergistic antitumor activity from Mn nanozymes, ELE, and DOP; (2) EpCAM-targeted delivery via CAR-engineered membranes; (3) retained catalytic activity of Mn nanozymes post-modification; (4) modulation of the immunosuppressive TME; and (5) broad-spectrum antibacterial and pro-healing action from quaternary ammonium salts and DOP. This integrated platform presents a promising strategy for addressing both tumor recurrence and wound infection after surgery.

## Supplementary Information

Below is the link to the electronic supplementary material.

Supplementary Material 1

## Data Availability

No datasets were generated or analysed during the current study.
