# Supplementary material for: Dual-functional cationic hydrogel engineered for simultaneous prevention of postoperative tumor recurrence and wound infection
Source: J Nanobiotechnology. 2026 Apr 29;24:575. doi: 10.1186/s12951-026-04490-3 (PMC13274178; doi:10.1186/s12951-026-04490-3)
Supplement: Supplementary file 1 — Supplementary Material 1 [file 12951_2026_4490_MOESM1_ESM.docx]

**Supporting information**

**Dual-Functional Cationic Hydrogel Engineered for Simultaneous Prevention of Postoperative Tumor Recurrence and Wound Infection**

Yating Qin^1*^, Ke Yao^1^, Yan Lin^1^, Xinyue Li^1^, Yifan Liu^1^, Yilin Li^1^, Yaping Li^2*^, Shuling Wang^1*^

*^1^**School of Pharmacy,* *Hangzhou Normal University, Hangzhou, 311121, China*

*^2^College of Chemical and Biological Engineering, Zhejiang University, Hangzhou, 311121, China.*

*Corresponding authors:*

*Shuling Wang (E-mail: wsling222@163.com);*

*Yating Qin (E-mail: qyt@hznu.edu.cn);*

*Yaping Li (E-mail: liyaping@zju.edu.cn).*

**Supporting** **experimental section.**

**Extraction and purification of *Dendrobium officinale* polysaccharide (DOP)**

The ultrafine powder of the dried stems of *Dendrobium officinale* was obtained from Zhejiang Qingbutang Biotechnology Co., Ltd. The extraction of DOP was based on our authorized Chinese invention patent titled "An Extraction Method for Active Components of *Dendrobium officinale*" (Patent No.: ZL 202010156053.8; Authorization Date: August 12, 2022). In brief, the extract of Dendrobium officinale obtained through hot water extraction was subjected to ethanol precipitation, followed by protein removal via treatment with trypsin and Sevag reagent (chloroform:n-butanol = 4:1, v/v). Subsequently, DOP were obtained through further purification involving activated carbon decolorization, dialysis, and freeze-drying. The purity of DOP was 99.82%.

**Measurement of catalase-like (CAT-like), peroxidase-like (POD-like) and oxidase-like (OXD-like) activities**

The CAT-like activity of CAR-M@Mn@ELE was investigated at 37 °C by measuring the oxygen generated using a dissolved oxygen meter. Five groups of 20 mL CAR-M@Mn@ELE solutions with varying concentrations were prepared using PBS at pH 5.5 as the solvent, followed by the addition of an equal volume of H_2_O_2_ solution (final concentration of 10 mM) to each group. The CAT-like activity of CAR-M@Mn@ELE catalyzes the decomposition of H_2_O_2_ into oxygen. The concentration dependence of this activity was assessed by measuring oxygen generation over a period of 0-10 min with a dissolved oxygen analyzer (JPSJ-605F, REX, China). Additionally, three groups of 20 mL CAR-M@Mn@ELE solutions (20 μg/mL) were prepared using PBS at different pH values as the solvent, to which an equal volume of H_2_O_2_ solution (final concentration of 10 mM) was added. A control group consisting of PBS at pH 5.5 (without CAR-M@Mn@ELE) supplemented with H_2_O_2_ (10 mM) was also established. The effect of varying pH on the CAT-like activity of CAR-M@Mn@ELE was evaluated by measuring oxygen generation over a period of 0-10 min using the dissolved oxygen analyzer. Furthermore, six groups of 20 mL H_2_O_2_ solutions (3, 5, 8, 10, 12 and 15 mM) were prepared using PBS at pH 6.5 as the solvent, with equal amounts of CAR-M@Mn@ELE (10 μg/mL) added to each. The oxygen generation during the first 0-2 min was monitored using a dissolved oxygen meter to conduct stability kinetic analysis of CAR-M@Mn@ELE. The Michaelis-Menten constant was determined from the Michaelis-Menten saturation curve.

The POD-like activity of CAR-M@Mn@ELE was measured at 37 °C using TMB as a probe. Six groups of 2 mL CAR-M@Mn@ELE solutions with varying concentrations were prepared using PBS buffer at pH 5.5 as the solvent. Equal amounts of H_2_O_2_ solution (10 mM) and TMB solution (1 mM) were subsequently added. The concentration dependent POD-like activity of CAR-M@Mn@ELE was assessed by recording the absorbance at 652 nm using a UV-vis spectrophotometer after a 4 min reaction. Three groups of 2 mL CAR-M@Mn@ELE solution (20 μg/ml) were prepared with PBS at different pH as the solvent, to which equal amounts of H_2_O_2_ (10 mM) and TMB (1 mM) were added. Additionally, a control group consisting of a pH 5.5 PBS buffer (without CAR-M@Mn@ELE) with H_2_O_2_ (10 mM) and TMB (1 mM) was included. After a 4 min reaction, the absorbance at 652 nm was recorded. Six groups of 2 mL CAR-M@Mn@ELE solutions (10 μg/mL) were prepared with PBS at pH 6.5, followed by the addition of equal amounts of TMB (1 mM) and varying amounts of H_2_O_2_ (10, 20, 30, 40, 50, and 60 mM) to each group. After 4 min, the absorbance of the product at 652 nm was recorded. The absorbance measurements were subsequently transformed into the concentration of oxidation products derived from TMB by employing the Beer-Lambert Law. The Michaelis-Menten constant was established based on the saturation curve of Michaelis-Menten.

The OXD-like activity of CAR-M@Mn@ELE was assessed at 37°C using TMB as both the probe and substrate. Six groups of 2 mL CAR-M@Mn@ELE solutions with varying concentrations were prepared in PBS at pH 5.5. An equal volume of TMB (1 mM) was added to each group. Following an 80 s reaction period, the absorbance at 652 nm was measured using a UV-vis spectrometer. The concentration-dependent OXD-like activity of CAR-M@Mn@ELE was evaluated based on changes in absorbance. Additionally, 2 mL of CAR-M@Mn@ELE solution (20 μg/mL) was prepared in PBS at different pH values, with the same TMB concentration (1 mM) added to each group. PBS (without CAR-M@Mn@ELE) at pH 5.5 and TMB concentration of 1 mM served as the control group. After an 80 s reaction, the absorbance at 652 nm was again measured. Equal volumes of CAR-M@Mn@ELE (10 μg/mL) were introduced to PBS (pH = 6.5) at varying TMB concentrations (10, 20, 40, 60, 80 and 100 μM) to ascertain the steady-state kinetics of CAR-M@Mn@ELE. Following an 80 s reaction, the absorbance of the product at 652 nm was monitored. The Michaelis-Menten constant was determined using the same methodology as that applied in the steady-state kinetic assessment of POD-like activity.

***In vitro* release of CAR-M@Mn@ELE from Gel@CAR-M@Mn@ELE**

To evaluate the *in vitro* release of CAR-M@Mn@ELE from Gel@CAR-M@Mn@ELE, experiments were conducted under conditions mimicking the postoperative microenvironment (PME-mimic) and control conditions. A PME-mimicking PBS solution (pH 6.5 with 0.8 μg/mL type I collagenase) and control PBS (pH 7.4) were used as release media. Briefly, 200 mg of Gel@CAR-M@Mn@ELE was placed in 20 mL of release medium and incubated in a shaking incubator at 37 °C. At predetermined time points, aliquots of the medium were collected and replaced with fresh medium. The collected samples were centrifuged, followed by digestion with concentrated nitric acid. The Mn content was then measured using ICP-MS to quantify the release and construct the release profile. Furthermore, to evaluate whether CAR-M@Mn@ELE retains its targeting ability after release from Gel@CAR-M@Mn@ELE, FITC-labeled CAR-M@Mn@ELE was incorporated into the hydrogel. Following 72 h of incubation in postoperative microenvironment-mimicking PBS, the released nanoparticles were collected and co-incubated with 4T1 and MDA-MB-231 cells, respectively. Targeting ability was subsequently analyzed by flow cytometry.

**Safety evaluation of** **Gel@CAR-M@Mn@ELE**

In vitro biosafety assessment of Gel@CAR-M@Mn@ELE was conducted using L929 cells. L929 cells were seeded into 96-well plates at a density of 1×10⁴ cells per well and incubated overnight under standard culture conditions. Following incubation, the culture medium was replaced with fresh medium containing extracts of Gel@CAR-M@Mn@ELE at varying concentrations (50-500 μg/mL), and the cells were co-incubated for an additional 24 h. Cell viability was subsequently evaluated using the CCK-8 assay. We mixed the diluted fresh blood of mice with the extract of Gel@CAR-M@Mn@ELE, and then incubated, centrifuged and determined the absorbance. At the same time, negative control groups (treated with normal saline) and positive control groups (treated with deionized water) were set up to evaluate the hemolytic property of the material. To further assess the in vivo safety profile of Gel@CAR-M@Mn@ELE, healthy mice were anesthetized and subcutaneously implanted with the material. On day 11, whole blood samples were collected for hematological analysis, and five major organs were harvested for histopathological evaluation via H&E staining.

***In vitro* antibacterial detection of Gel@CAR-M@Mn@ELE**

The colony counting method was used to assess the antibacterial effectiveness of different hydrogels on gram-positive *S. aureus* and gram-negative *E. coli*. Initially, different hydrogels were prepared in 48-well plates, followed by the addition of 200 µL of *S. aureus* or *E. coli* suspension (10^8^ CFU/mL) which were then incubated at 37℃ for 8 h. Groups: Normal saline as control group, Gel without DOP group, Gel and Gel@CAR-M@Mn@ELE group. Subsequently, the bacterial stock solutions from each group were diluted 10^-6^ times, and 5 μL of bacterial was taken and incubated on the LB agar plates for 20 h at 37℃. The colonies from each group were photographed and counted. The antimicrobial rate (AR) was calculated as follows: AR (%) = (N_C_-N_S_)/N_C_×100%, in which N_C_ denotes the bacterial colonies present in the control sample and N_S_ indicates the bacterial colonies found in the hydrogel sample. To further evaluate the antibacterial effect, live/dead bacterial staining was utilized. Each group underwent treatment as previously outlined. Then, the bacteria were stained according to the instructions provided by the DMAO/PI kit. The staining results were examined by CLSM. Additionally, SEM was utilized to observe morphological alterations in *S. aureus* subjected to various treatments.

**Supporting figures.**

**
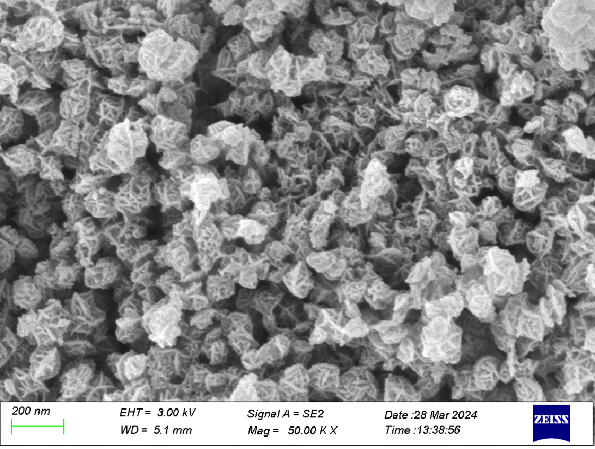
**

**Figure S1.** The SEM of Mn NPs.

**
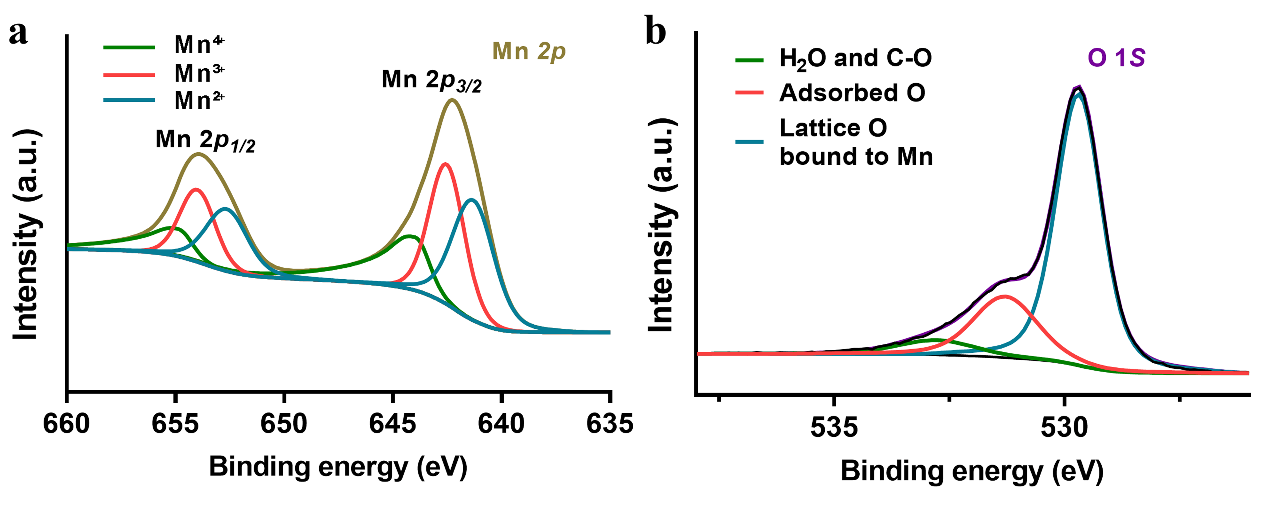
**

**Figure S2.** (a) Mn 2p XPS spectrum for Mn NPs. (b) O 1s XPS spectrum for Mn NPs.


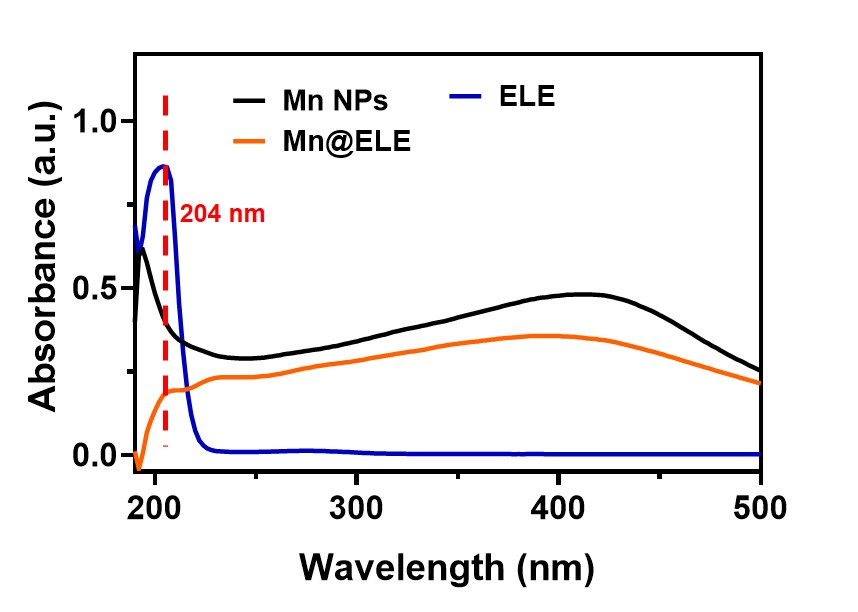


**Figure S3.** UV-vis spectra of the Mn NPs, ELE and Mn@ELE.

**
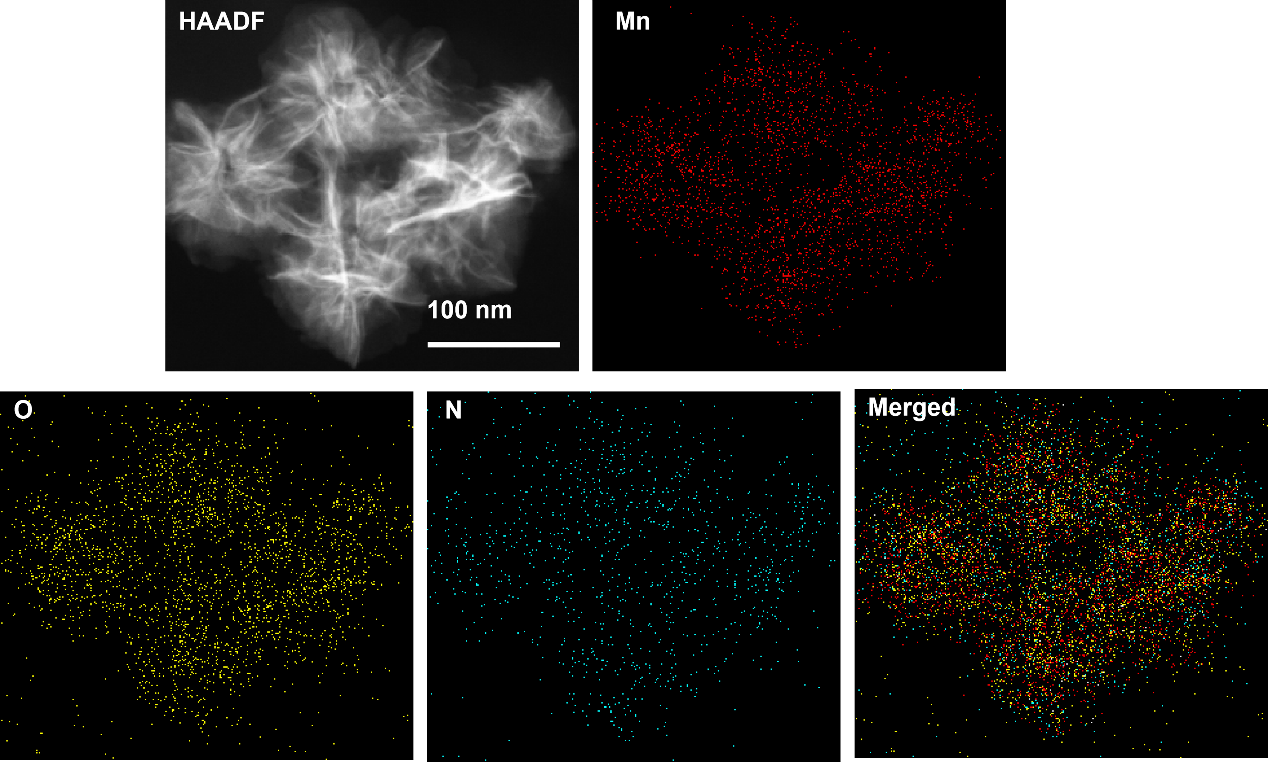
**

**Figure S4.** The STEM and corresponding elemental mapping of CAR-M@Mn@ELE.

**
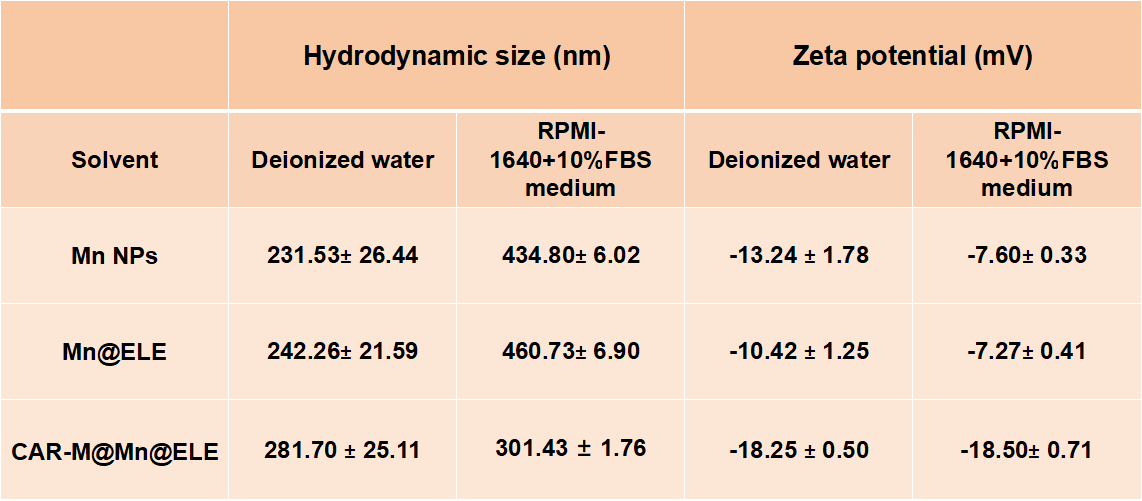
**

**Figure S5.** The hydrodynamic size and zeta potential of Mn NPs, Mn@ELE and CAR-M@Mn@ELE (Concentration: 0.125 mg/ml) in deionized water and RPMI-1640+10% FBS medium, respectively.


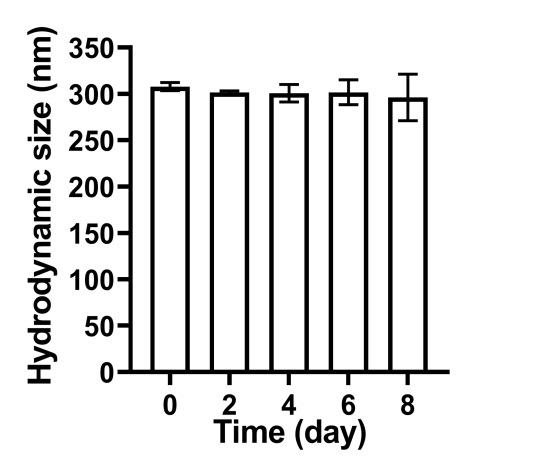


**Figure S6.** The hydrodynamic size of CAR-M@Mn@ELE (Concentration: 0.125 mg/ml) at different times in RPMI-1640+10% FBS medium.

**
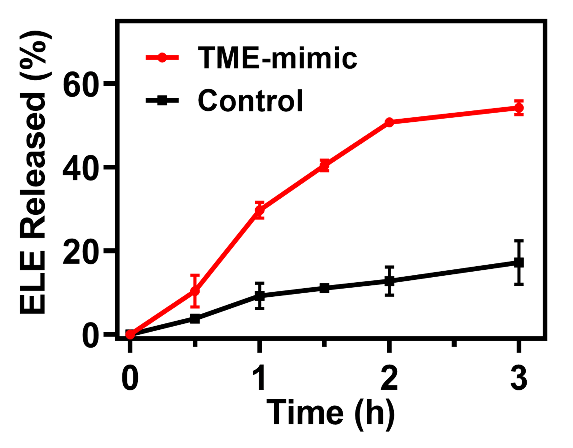
**

**Figure S7.** Accumulated release profile of ELE from CAR-M@Mn@ELE in different conditions (TME-mimic: pH 6.5 with 2 mM GSH, 100 μM H_2_O_2_ and 0.5% SDS; Control: pH 7.4 with 0.5% SDS).

**
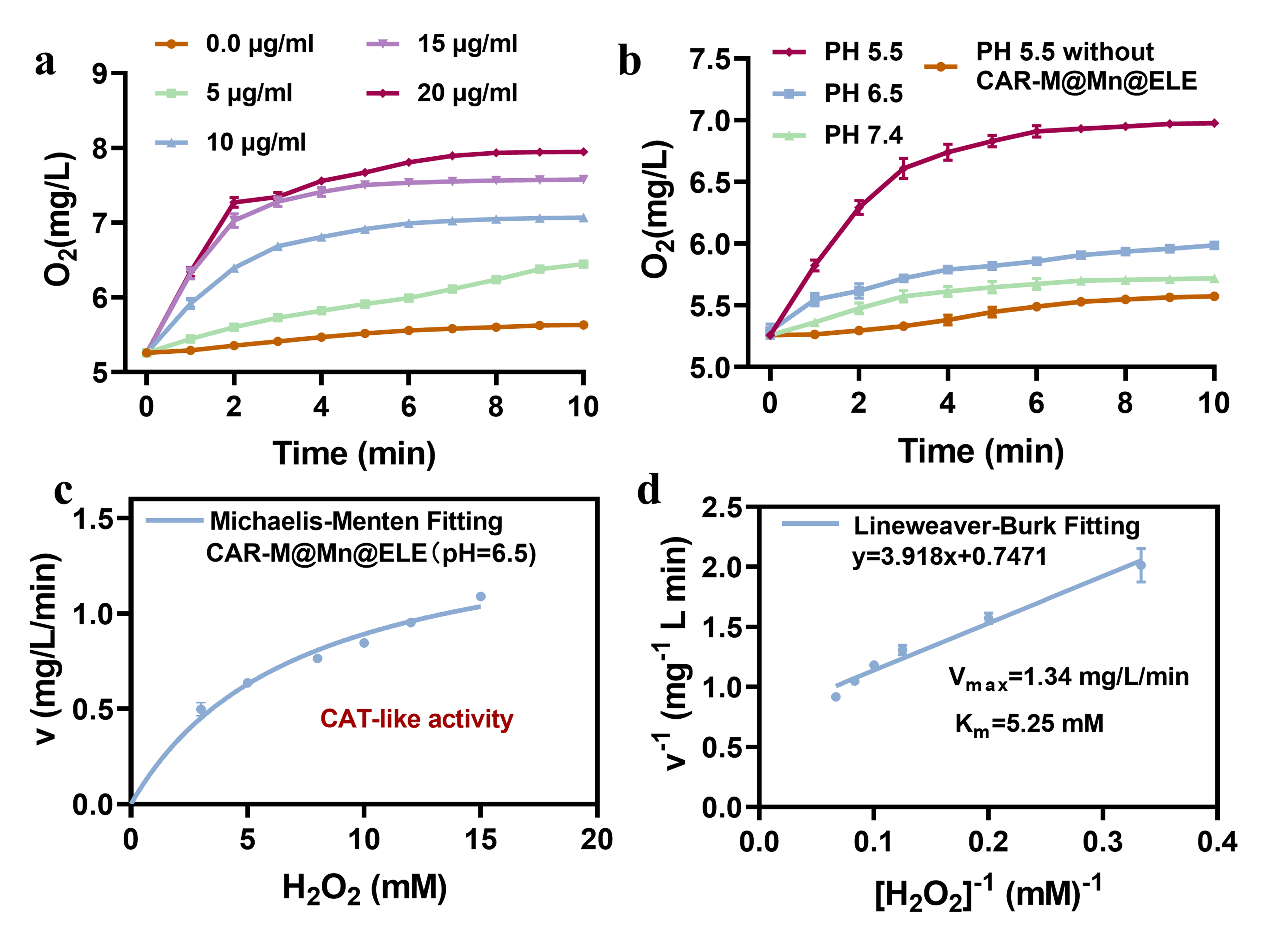
**

**Figure S8.** (a) The amount of O_2_ produced by catalyzing the same amount of H_2_O_2_ with different concentrations of CAR-M@Mn@ELE in an acidic environment (pH5.5). (b) The amount of O_2_ produced by catalyzing the same concentration of H_2_O_2_ with the same amount of CAR-M@Mn@ELE at different pH values. CAT-like activity-related Michaelis-Menten kinetic analysis (c) and Lineweaver-Burk plot (d) for CAR-M@Mn@ELE with H_2_O_2_ as a substrate.

**
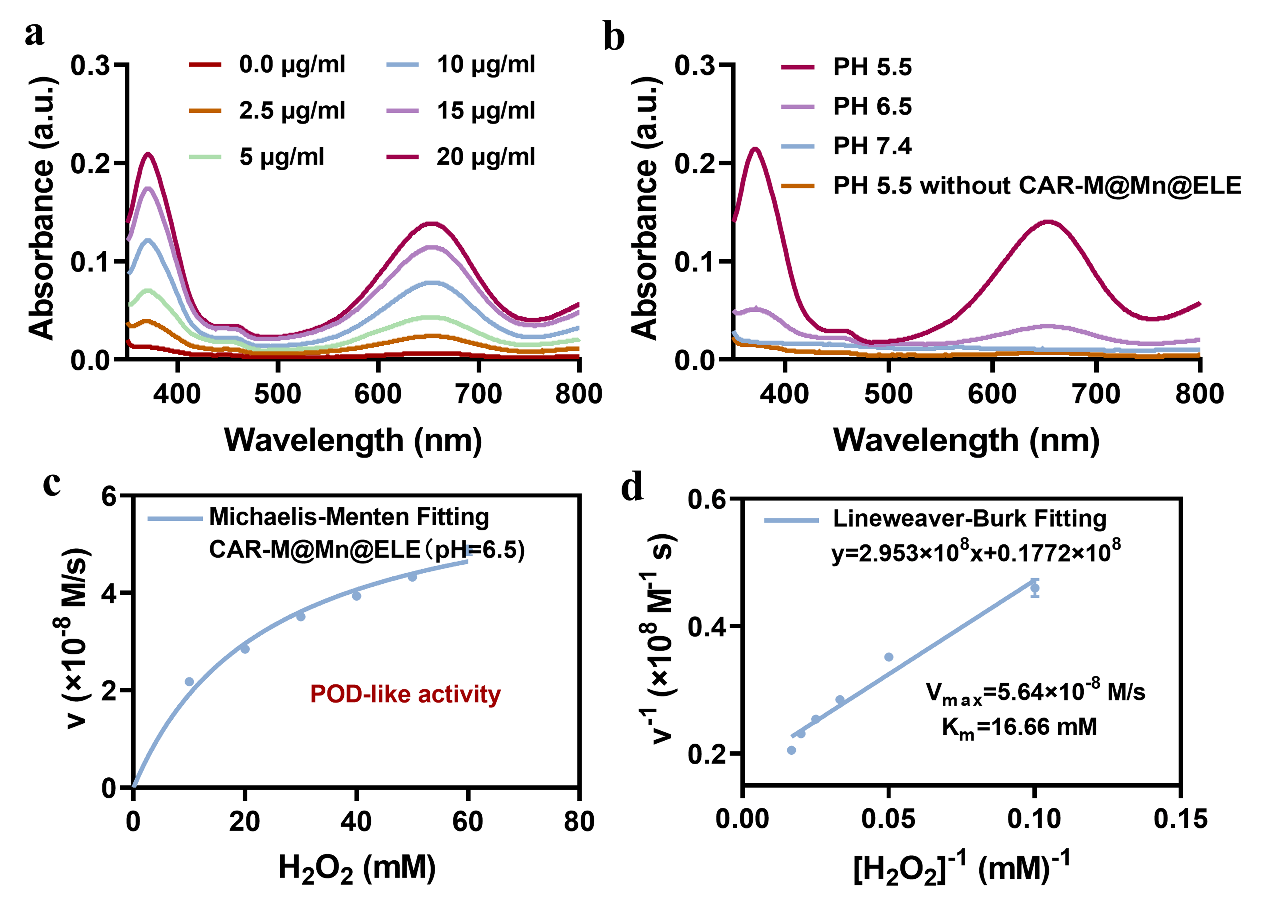
**

**Figure S9.** (a) UV-vis spectra of oxTMB in an acidic environment (pH5.5) with the addition of H_2_O_2_ and different concentrations of CAR-M@Mn@ELE. (b) UV-vis spectra of oxTMB at different pH conditions with the addition of the same concentrations of H_2_O_2_ and CAR-M@Mn@ELE. POD-like activity-related Michaelis-Menten kinetic analysis (c) and Lineweaver-Burk plot (d) for CAR-M@Mn@ELE with H_2_O_2_ as a substrate.

**
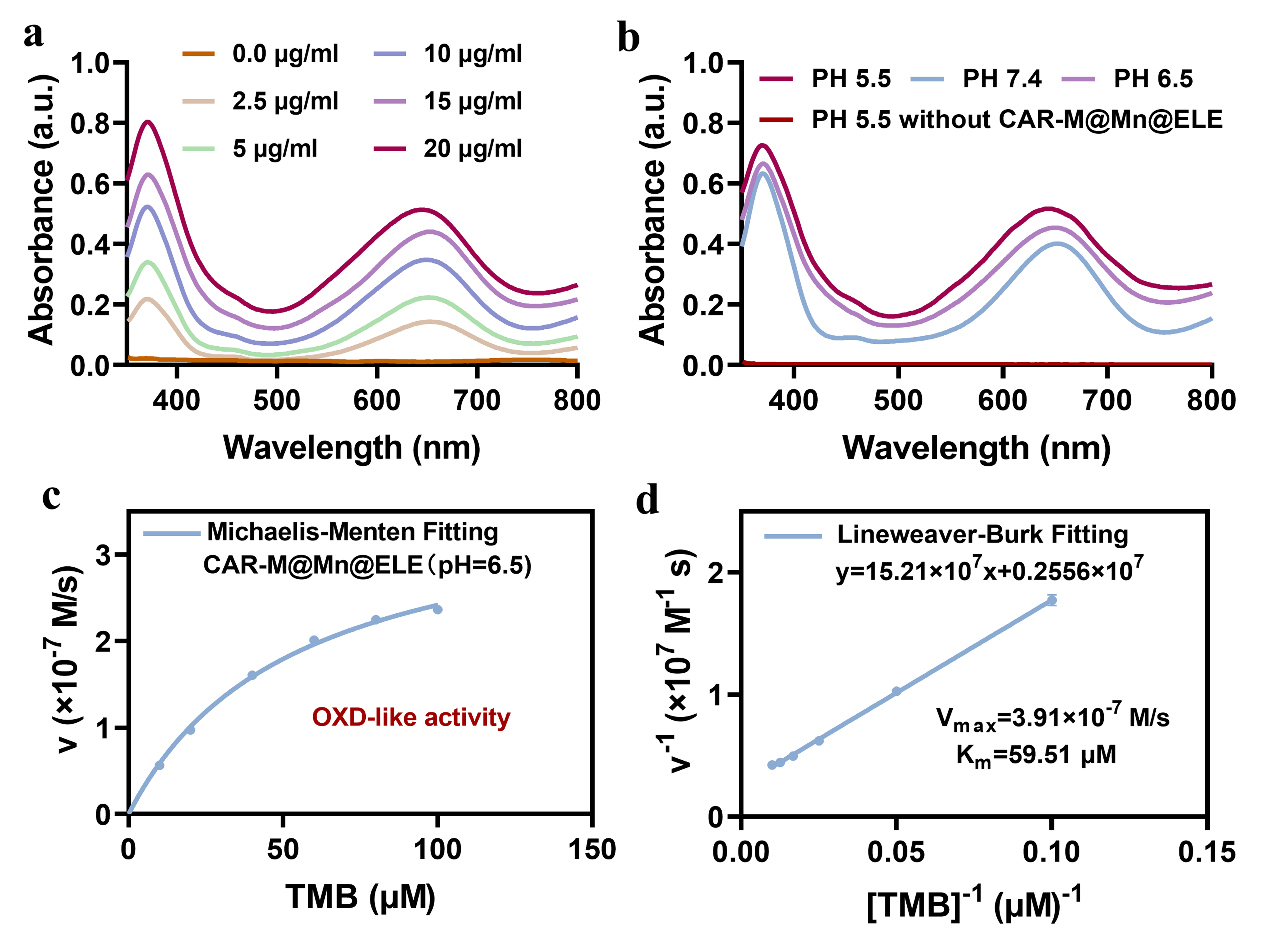
**

**Figure S10.** (a) UV-vis spectra of oxTMB upon the addition of different concentrations of CAR-M@Mn@ELE (pH 5.5). (b) UV-vis spectra of oxTMB upon the addition of CAR-M@Mn@ELE with different pH values, pH 5.5 without CAR-M@Mn@ELE as a control. OXD-like activity-related Michaelis-Menten kinetic analysis (c) and Lineweaver-Burk plot (d) for CAR-M@Mn@ELE with TMB as a substrate.

**
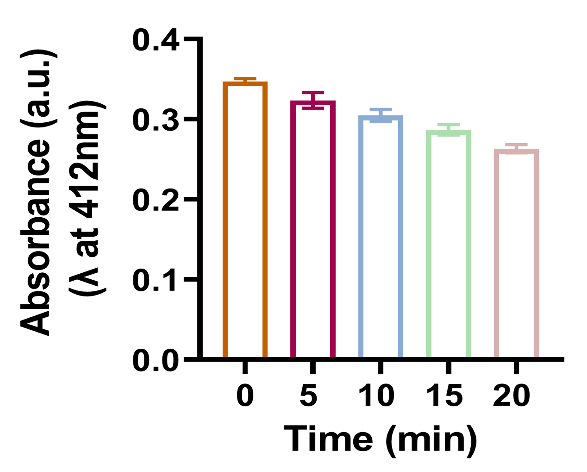
**

**Figure S11.** Time-dependent GSH consumption by CAR-M@Mn@ELE.


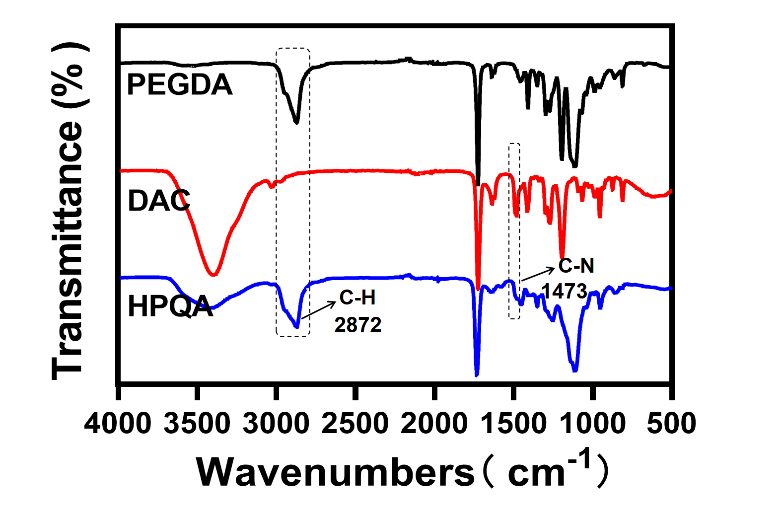


**Figure S12.** ATR-IR profiles of PEGDA, DAC and HPQA.

**
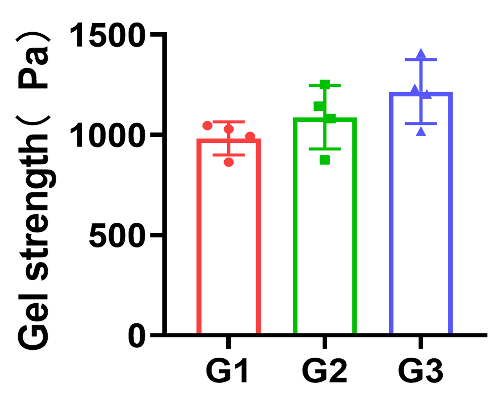
**

**Figure S13.** The gel strength of G1 (Gel without DOP), G2 (Gel) and G3 (Gel with DOP and CAR-M@Mn@ELE).


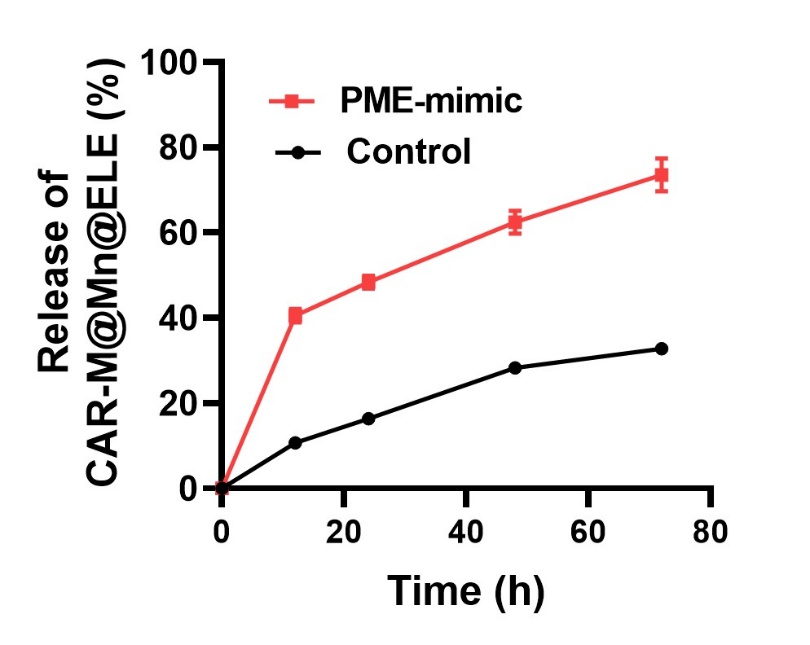


**Figure S14.** The release curves of CAR-M@Mn@ELE from Gel@CAR-M@Mn@ELE under different conditions (PME-mimic: pH 6.5 with 0.8 μg/mL type I Collagenase; Control: pH 7.4).


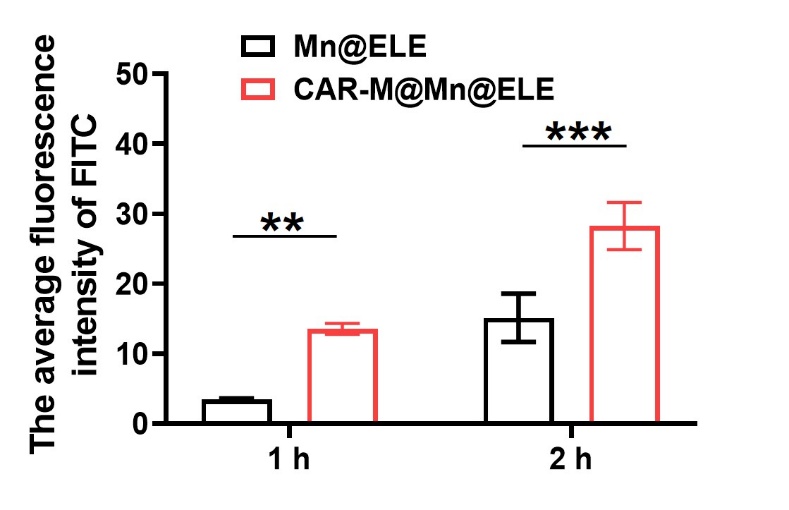


**Figure S15.** The statistical analysis results of Figure 3a. The data represent the mean ± SD, n=3. **P < 0.01 and ***P < 0.001.


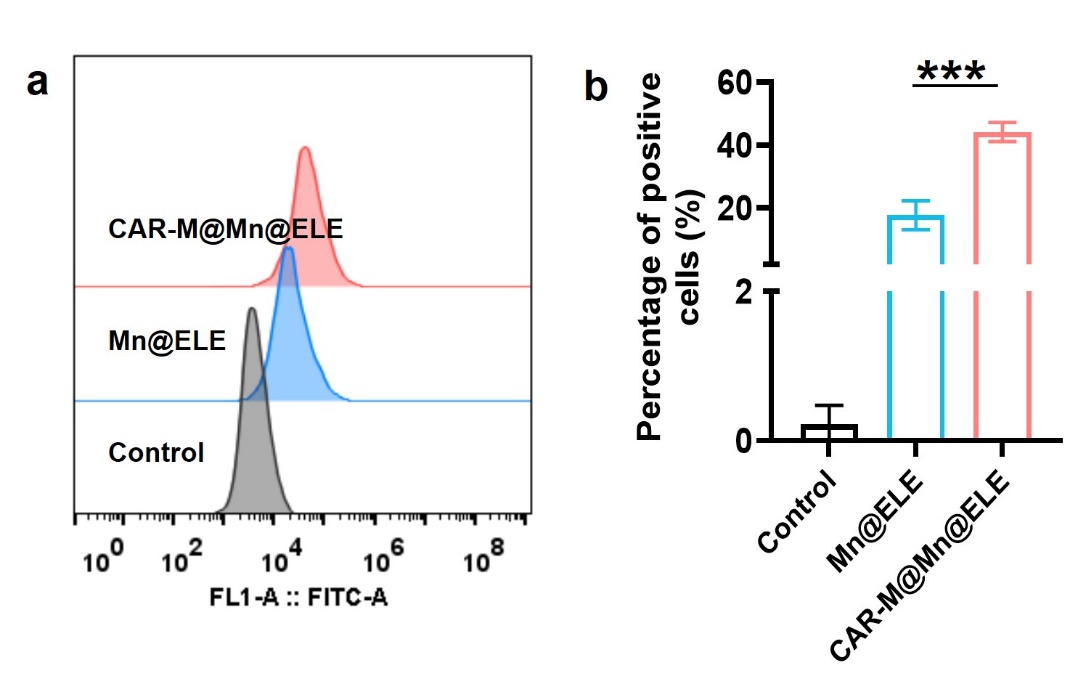


**Figure S16.** Representative flow cytometry plots (a) and corresponding quantitative analysis of positive cell rate (b) of 4T1 cells after co-incubation with Mn@ELE or CAR-M@Mn@ELE for 2 h, respectively. The data represent the mean ± SD, n=3. ****P<0.0001.


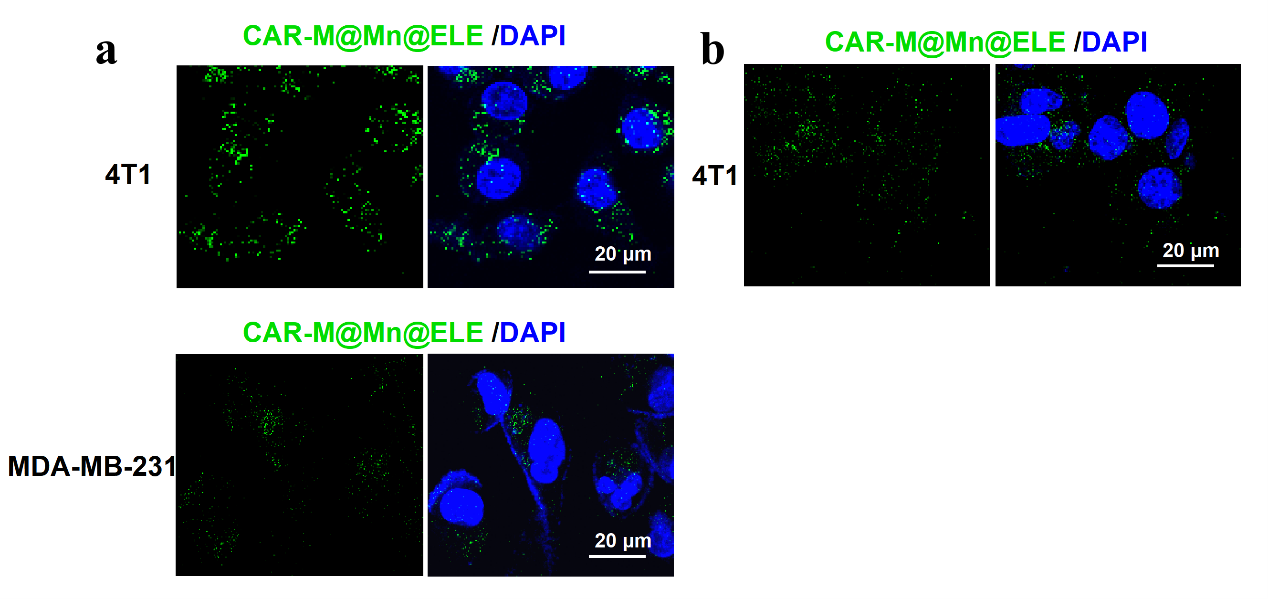


**Figure S17**. (a) CLSM images of 4T1 and MDA-MB-231 cells after co-incubation with CAR-M@Mn@ELE for 1 h, respectively. (b) Confocal laser microscopy images of 4T1 cells after co-incubation with anti-EpCAM antibody and then co-incubation with CAR-M@Mn@ELE for 1 h.


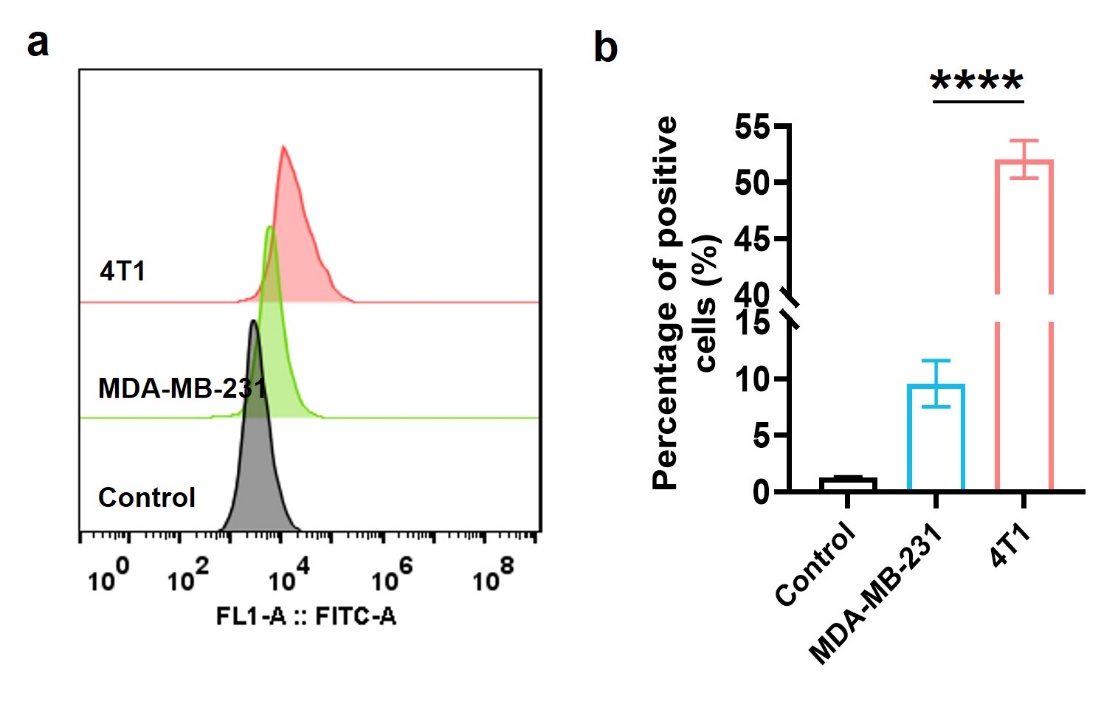


**Figure S18.** Representative flow cytometry plots (a) and corresponding quantitative analysis of positive cell rate (b) of 4T1 and MDA-MB-231 cells after co-incubation with CAR-M@Mn@ELE released from the Gel@CAR-M@Mn@ELE for 2 h. The data represent the mean ± SD, n=3. ****P<0.0001.

**
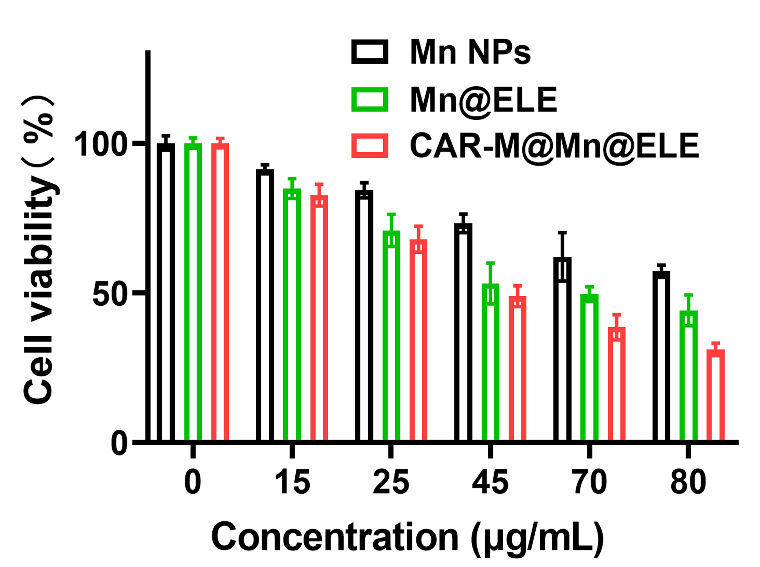
**

**Figure S19.** Viability of 4T1 cells treated with different concentrations of Mn NPs, Mn@ELE and CAR-M@Mn@ELE for 24 h.


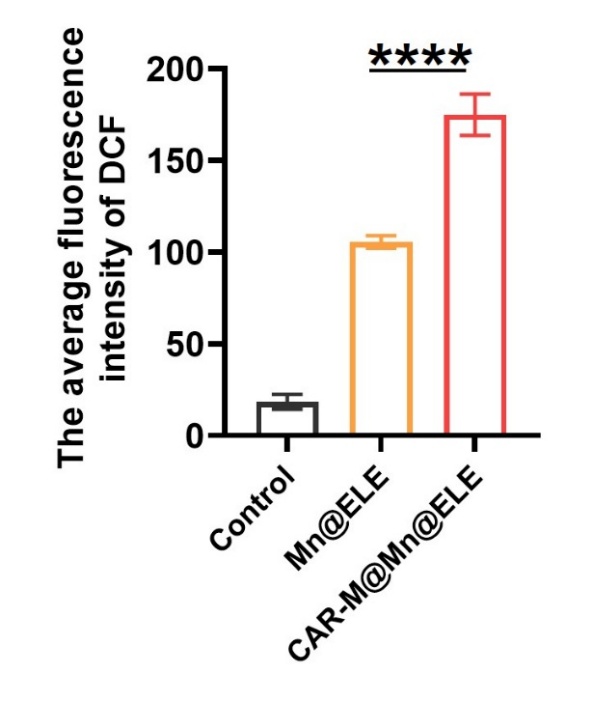


**Figure S20.** The statistical analysis results of Figure 3c. The data represent the mean ± SD, n=3. ****P<0.0001.


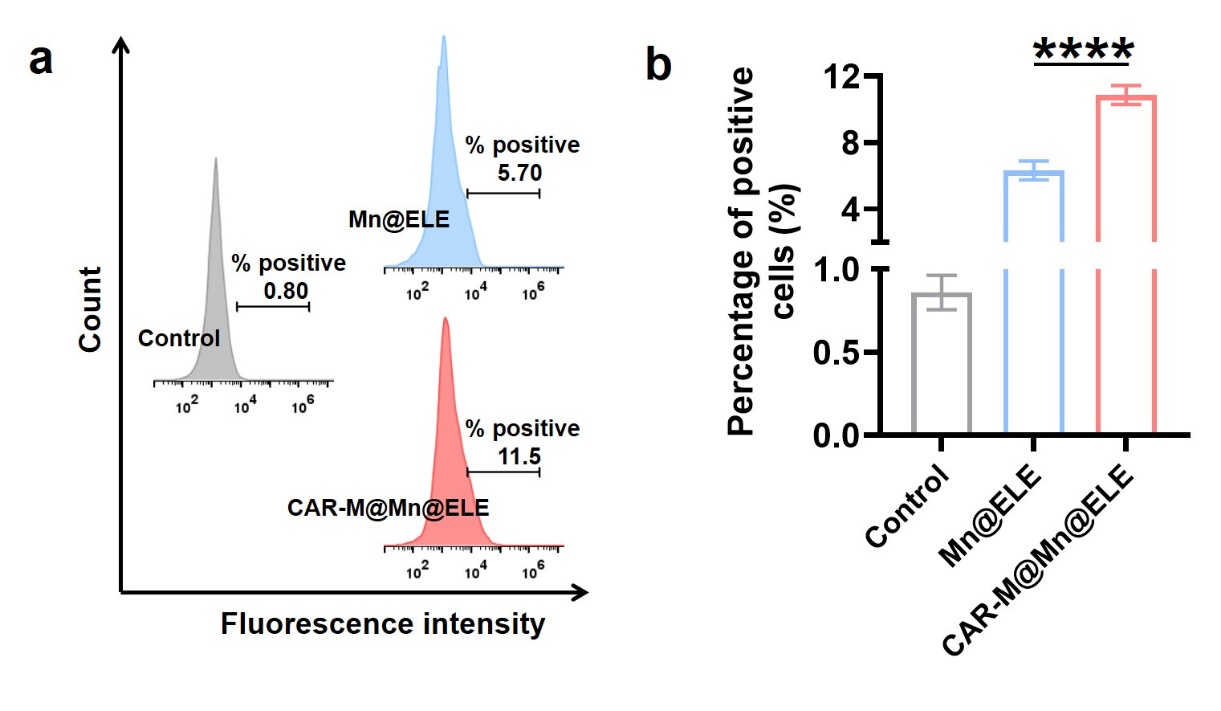


**Figure S21.** Representative flow cytometry plots (a) and corresponding quantitative analysis of positive cell rate (b) of intracellular ROS levels in 4T1 cells after co-incubation with Mn@ELE and CAR-M@Mn@ELE, respectively. The data represent the mean ± SD, n=3. ****P<0.0001.

**
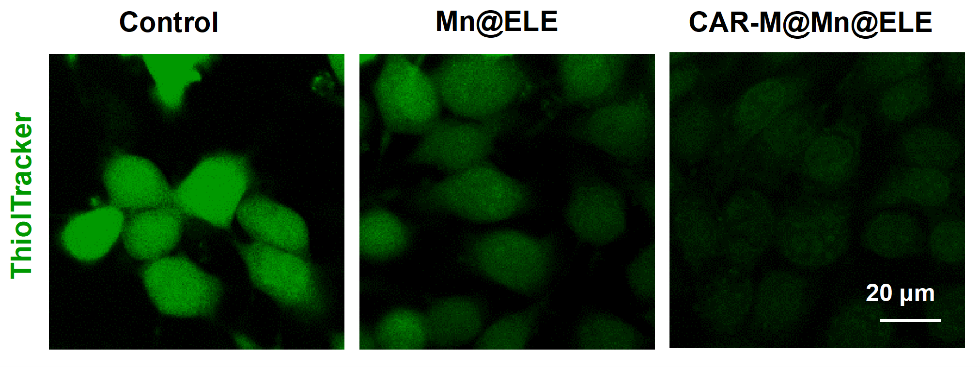
**

**Figure S22.** CLSM images of intracellular GSH after treatment with Mn@ELE and CAR-M@Mn@ELE (ThiolTracker used as the probe).


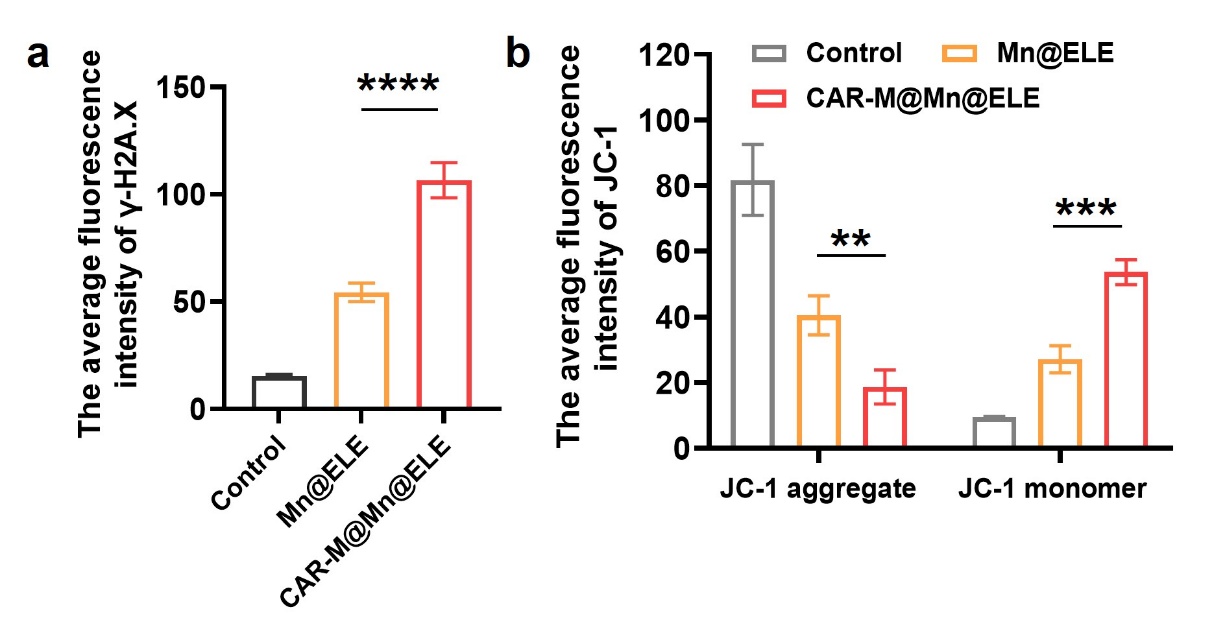


**Figure S23.** The statistical analysis results of Figure 3d (a) and 3e (b). The data represent the mean ± SD, n=3. **P < 0.01, ***P < 0.001 and ****P<0.0001.

**
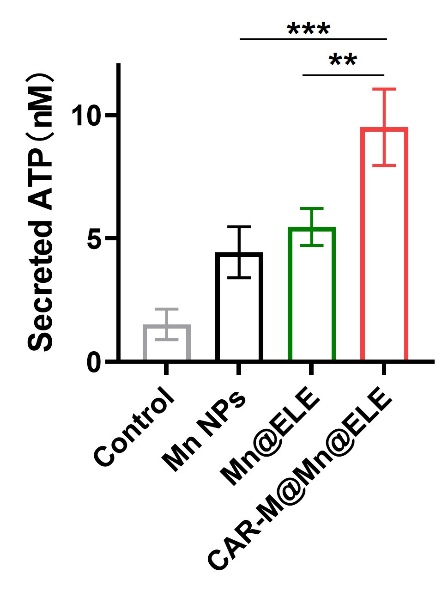
**

**Figure S24.** Quantitative analysis of released ATP in the medium after treatment with Mn NPs, Mn@ELE and CAR-M@Mn@ELE for 12 h. The data represent the mean ± SD, n=3. **P < 0.01 and ***P < 0.001.

**
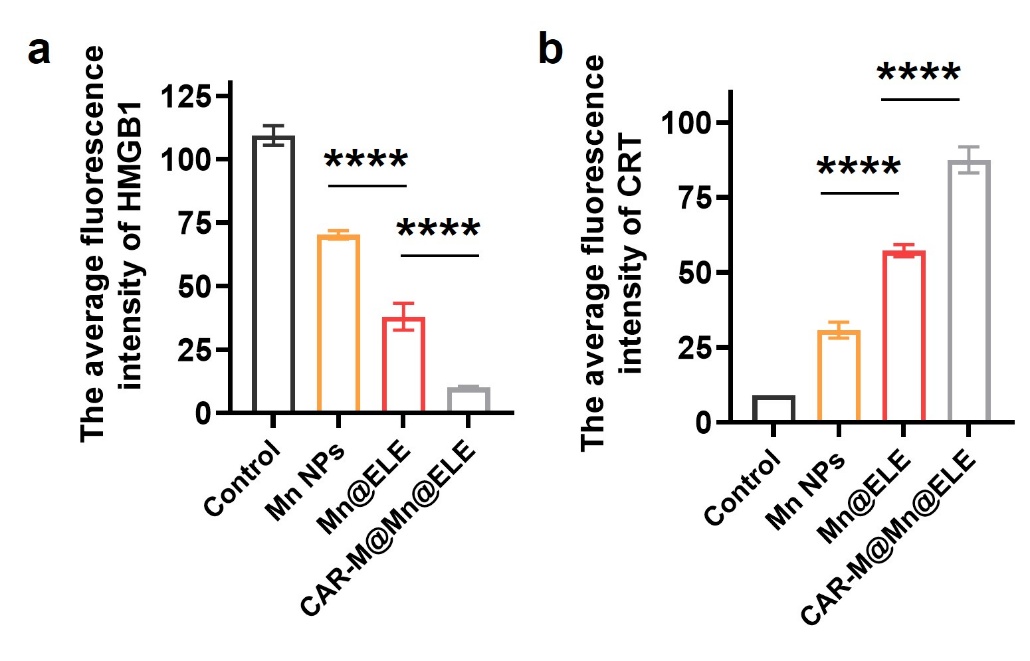
**

**Figure S25.** The statistical analysis results of Figure 3f (a) and 3g (b). The data represent the mean ± SD, n=3. ****P<0.0001.


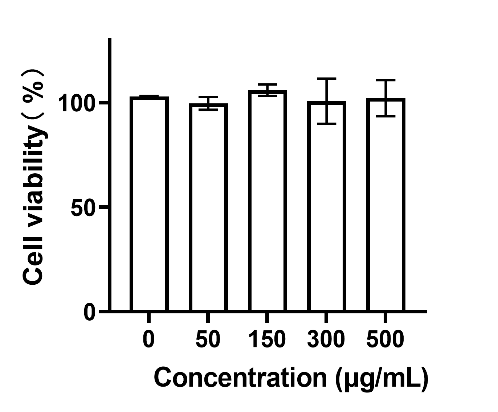


**Figure S26.** Viability of L929 cells treated with different concentrations of extracting solution of Gel@CAR-M@Mn@ELE (50-500 μg/mL) for 24 h.

**
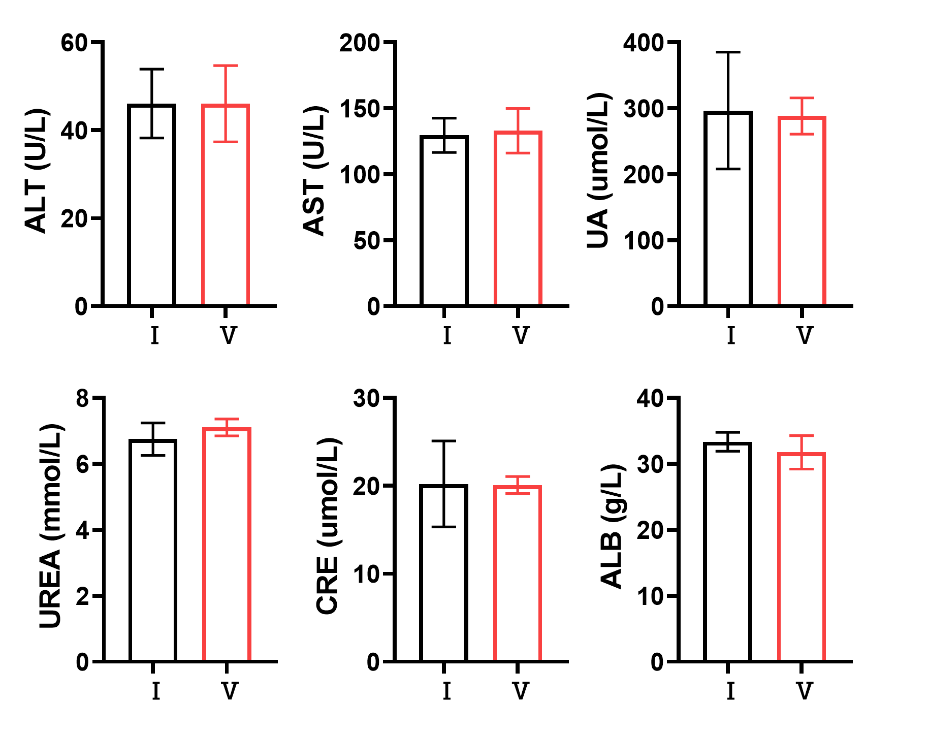
**

**Figure S27.** Various blood biochemical parameters in blood samples of Gel@CAR-M@Mn@ELE treated mice at day 11; Healthy mice without treatment were employed as the control group. Ⅰ (Control), Ⅴ (Gel@CAR-M@Mn@ELE). ALT: alanine aminotransferase, AST: aspartate aminotransferase, UA: uric acid, UREA: urea, CRE: creatinine, ALB: albumin.


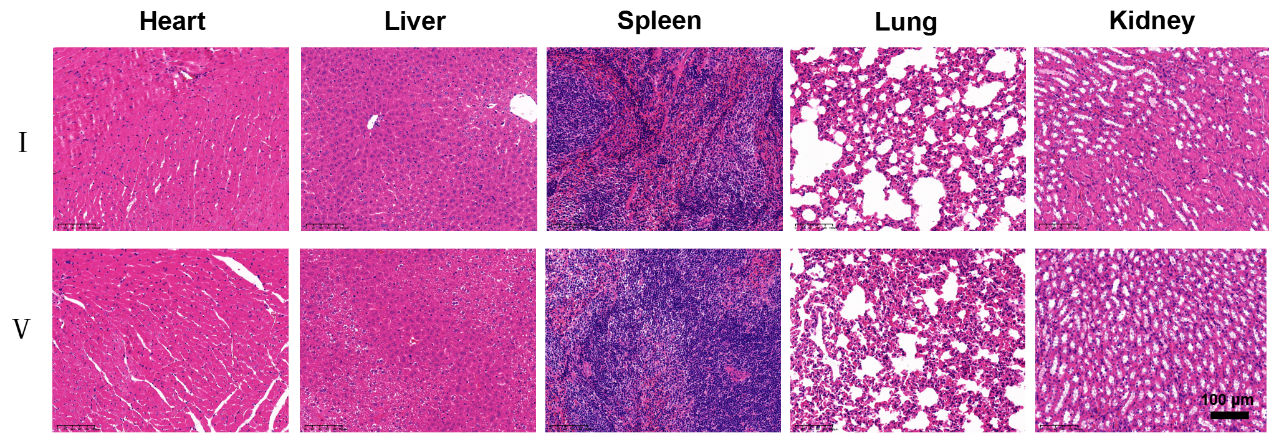


**Figure S28.** H&E staining micrographs of major organs (heart, liver, spleen, lung, and kidney) obtained from healthy mice and mice treated with Gel@CAR-M@Mn@ELE on day 11. Ⅰ (Control), Ⅴ (Gel@CAR-M@Mn@ELE). Scale bar: 100 μm.


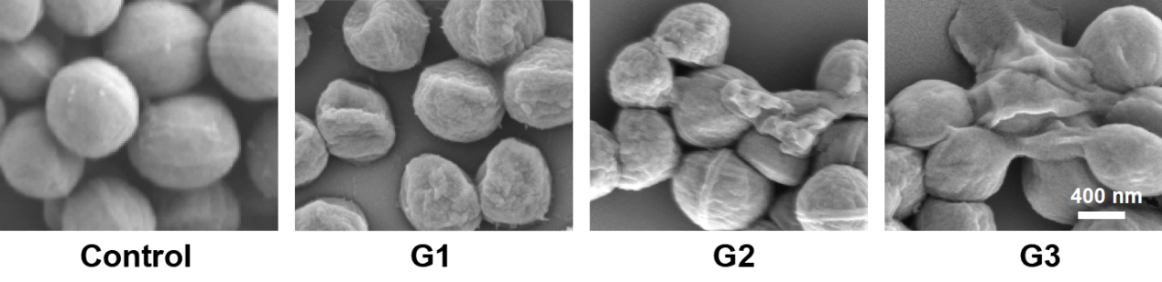


**Figure S29.** SEM images of *S. aureus* after different treatments. Control (Normal saline), G1 (Gel without DOP), G2 (Gel) and G3 (Gel with DOP and CAR-M@Mn@ELE). Scale bar: 400 nm.


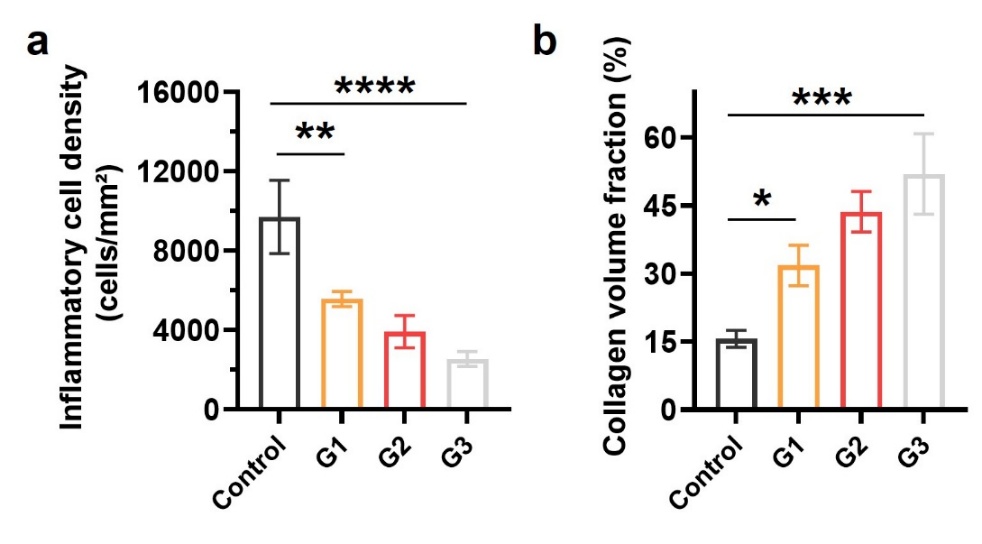


**Figure S30.** (a) Quantitative analysis of inflammatory cell density in Figure 7f. (b) Quantitative analysis of the collagen volume fraction in Figure 7g. The data represent the mean ± SD, n=3. *P<0.05, **P < 0.01, ***P < 0.001 and ****P<0.0001.
